# A compound directed against S6K1 hampers fat mass expansion and mitigates diet-induced hepatosteatosis

**DOI:** 10.1172/jci.insight.150461

**Published:** 2022-07-22

**Authors:** Aina Lluch, Sonia R. Veiga, Jèssica Latorre, José M. Moreno-Navarrete, Núria Bonifaci, Van Dien Nguyen, You Zhou, Marcus Höring, Gerhard Liebisch, Vesa M. Olkkonen, David Llobet-Navas, George Thomas, Ruth Rodríguez-Barrueco, José M. Fernández-Real, Sara C. Kozma, Francisco J. Ortega

**Affiliations:** 1 Bellvitge Biomedical Research Institute (IDIBELL), L’Hospitalet de Llobregat, Barcelona, Spain.; 2 Girona Biomedical Research Institute (IDIBGI), Girona, Spain.; 3 CIBER Fisiopatología de la Obesidad y Nutrición (CIBEROBN), and Institute of Salud Carlos III (ISCIII), Madrid, Spain.; 4 Systems Immunity Research Institute, Cardiff University, Cardiff, United Kingdom.; 5 Division of Infection and Immunity, Cardiff University School of Medicine, Cardiff, United Kingdom.; 6 Institute of Clinical Chemistry and Laboratory Medicine, Regensburg University Hospital, Regensburg, Germany.; 7 Minerva Foundation Institute for Medical Research, Biomedicum 2U, Helsinki, Finland.; 8 Department of Anatomy, Faculty of Medicine, University of Helsinki, Helsinki, Finland.; 9 Institute of Genetic Medicine, Newcastle University, Newcastle, United Kingdom.; 10 School of Medicine, University of Barcelona (UB), Barcelona, Spain.

**Keywords:** Metabolism, Therapeutics, Adipose tissue, Obesity, Pharmacology

## Abstract

The ribosomal protein S6 kinase 1 (S6K1) is a relevant effector downstream of the mammalian target of rapamycin complex 1 (mTORC1), best known for its role in the control of lipid homeostasis. Consistent with this, mice lacking the *S6k1* gene have a defect in their ability to induce the commitment of fat precursor cells to the adipogenic lineage, which contributes to a significant reduction of fat mass. Here, we assess the therapeutic blockage of S6K1 in diet-induced obese mice challenged with LY2584702 tosylate, a specific oral S6K1 inhibitor initially developed for the treatment of solid tumors. We show that diminished S6K1 activity hampers fat mass expansion and ameliorates dyslipidemia and hepatic steatosis, while modifying transcriptome-wide gene expression programs relevant for adipose and liver function. Accordingly, decreased mTORC1 signaling in fat (but increased in the liver) segregated with defective epithelial-mesenchymal transition and the impaired expression of Cd36 (coding for a fatty acid translocase) and Lgals1 (Galectin 1) in both tissues. All these factors combined align with reduced adipocyte size and improved lipidomic signatures in the liver, while hepatic steatosis and hypertriglyceridemia were improved in treatments lasting either 3 months or 6 weeks.

## Graphical abstract

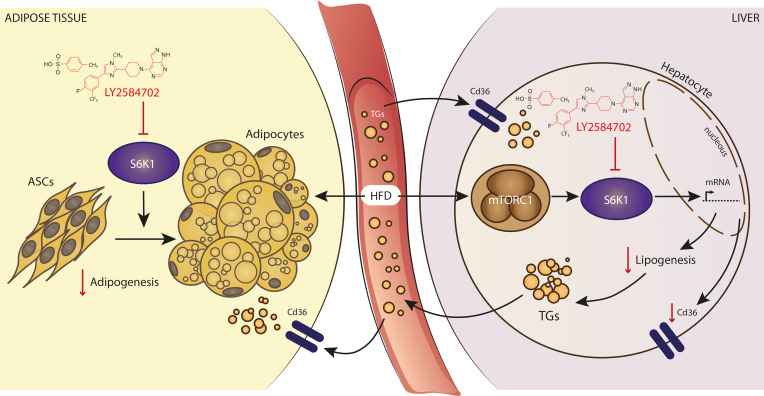

## Introduction

Liver and adipose tissue control body lipid homeostasis. Reciprocal dysfunction in these organs during sustained consumption of diets containing high amounts of fat may propel the cluster of metabolic disturbances associated with obesity (1). Among these, dyslipidemia — including both hypertriglyceridemia and hypercholesterolemia — constitutes a common hallmark of the obesity-related metabolic imbalance that may trigger a host of comorbidities, accounting for the so-called metabolic syndrome (2). Also, hepatic steatosis is a key pathogenic factor in perturbed lipid homeostasis, accelerating atherosclerosis and positioning dyslipidemia at the interface of obesity and the risk of cardiovascular and metabolic diseases (3–5). Thus, a pharmacological compound that lowers fat mass expansion and ameliorates lipid handling by the liver, protecting against both hepatic steatosis and dyslipidemia, would offer a great advancement for the treatment of metabolic syndromes related to the obese phenotype.

The ribosomal protein S6 kinase 1 (S6K1) operates downstream of the mammalian target of rapamycin complex 1 (mTORC1), which controls the response to hormones and mitogens, also coordinating cellular reactions to nutrients and energy inputs (6). The activation of S6K1 is mediated by an ordered series of conformational changes and phosphorylation steps, where T389 phosphorylation by mTORC1 creates a docking site for the phosphoinositide-dependent kinase 1 (PDK1), allowing T229 phosphorylation (7). This leads to S6K1 activation and its nuclear translocation (8). In turn, the nuclear translocation of S6K1 mediates the phosphorylation of Histone 2B (H2B) at S36 (H2BS36p), triggering the recruitment of the EZH2 methylase to H3 and trimethylation of H3 at K27 (H3K27me3). In fat cell precursors, this cascade represses the Wnt signaling pathway, causing the upregulation of PPARγ and CCAAT/enhancer-binding protein α (C/EBPα) (9). These events together allow the differentiation of mesenchymal stem cells into adipocytes, which are cells able to synthetize and store massive amounts of triglycerides (TGs) (10). Concurrently, the role that S6K1 displays in the regulation and maintenance of lipid homeostasis through the activation of Sterol regulatory element binding proteins (SREBPs) has been highlighted (11). Thus, through the activation of key transcription factors in liver (12) and adipose tissues (13), S6K1 can coordinate molecular changes of key relevance to the synthesis of fatty acids in cell systems where the homeostasis of lipids is of the utmost importance.

In the last years, the notion that imbalanced mTORC1-S6K1 engages signaling cascades related to impaired metabolism, obesity, fatty liver disease, and cancer has been disclosed (7, 14, 15). Our laboratory was the first to our knowledge to generate a S6K1-KO mouse model (16), which significantly contributed to the functional characterization of this kinase and revealed its importance in adipose development (17). This latter study also showed that S6K1 is a key regulator of the adipogenic capability and the distribution of energy surplus among newly formed adipocytes. Accordingly, *S6k1*-null mice exhibited a dramatic reduction in fat mass due to their reduced capacity to commit precursor cells to the adipocyte lineage (9, 13), thus limiting the expandability of fat depots. On the other hand, specific depletion of *S6k1* in liver appears to protect against diet-induced hepatosteatosis, as the induction of lipogenic genes was attenuated in liver-specific *S6k1*-deficient mice, leading to decreased steatosis even under conditions of high-fat diet (HFD) (18). Here, we present — for the first time to our knowledge in the obesity arena — in vivo, in vitro, functional, and mechanistic data that demonstrate the therapeutic utility of a compound directed against S6K1, the LY2584702 tosylate (hereafter referred to as LY), an oral inhibitor initially developed for the treatment of solid tumors (19). This drug increases phosphorylation of S6K1 (pS6K1) at T389 due to the competitive inhibition of ATP in molecular docking with the active site of S6K1 (20), thus blocking its kinase activity. We demonstrate that treatments with LY in mice challenged with a 60% HFD display decreased adiposity, as the result of a lower adipocyte size and gene expression patterns, suggestive of dampened energy storage in fat depots. In addition, these treatments diminished hepatic steatosis and modified transcriptomes in the liver parenchyma. Importantly, liver samples were also analyzed using untargeted lipidomics, providing lipid signatures as consistent insight into therapeutic changes affecting the saturated TGs content and increased phosphatidylinositols (PI), phosphatidylserines (PS), and phosphatidylcholines (PC), resembling the lipid signature of mice fed a normal chow (NC). Finally, we demonstrated that the beneficial effects of LY treatment on lipid homeostasis expands beyond the partial blockage of weight gain in high-fat and high-sucrose diet–induced obese mice. These findings highlight the potential of LY as an efficacious therapeutic agent to relieve the burden of dyslipidemia and fatty liver disease in obese patients.

## Results

### Treatments of LY protect against diet-induced obesity and liver steatosis.

**Figure 1:**
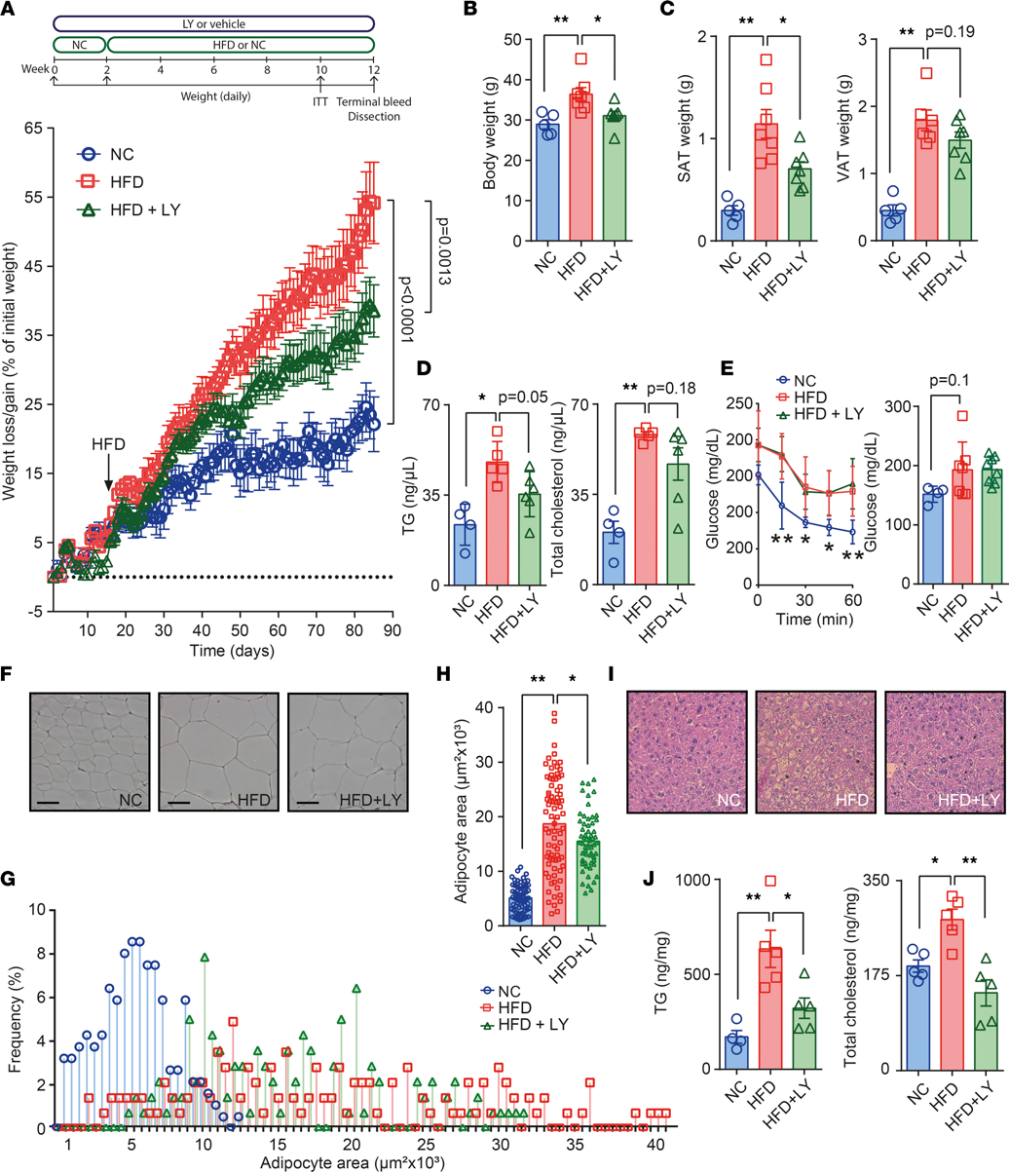
Physiological effects of LY on mice undergoing a high-fat diet. (**A**) Pipeline diagram for setting #1 and weight gain curve of mice under normal chow (NC) and high-fat diet (HFD) plus vehicle, and HFD plus oral gavage (25 mg/kg/12 h) with the S6K1 inhibitor LY2584702 tosylate (HFD + LY). (**B** and **C**) Body weight measures taken just before sacrifice, and weight of inguinal s.c. (SAT) and epididymal visceral (VAT) depots of white adipose tissue (*n* = 5/7/7 for NC, HFD, and HFD + LY, respectively). (**D** and **E**) Circulating triglycerides (TG) (**D**) and cholesterol at fasting (*n* = 4/5/6) and measures of glucose during an i.p. insulin tolerance test (ITT) and in 6-hour fasted mice (**E**) (*n* = 4/7/7). (**F**–**H**) Representative images of H&E staining in SAT, distribution of adipocyte sizes (%), and total adipocyte area in NC, HFD and HFD + LY mice (*n* = 2/3/3). Scale bar: 100 nm. (**I**) Representative 200× images of H&E staining in liver samples. (**J**) Quantification of the lipid content (TG and total cholesterol) of NC, HFD, and HFD + LY livers (*n* = 4/5/5). Data are presented as mean ± SEM using 2-way ANOVA followed by the Šidák’s correction, and adjusted *P* values for repeated measures are provided in weight gain curves (i.e., HFD versus NC, and HFD + LY versus HFD). The Dunnett’s multiple-comparison procedure was applied to the rest of measurements, in which nontreated HFD-fed mice served as the reference group. *P < 0.05, **P < 0.01.

We have previously reported that mice deficient for *S6k1* are protected against obesity and impaired metabolism (13, 17). Here, our primary objective was to assess the pharmacological effects of inhibiting S6K1 in adult mice subjected to diet-induced obesity (DIO). To characterize the ability of the S6K1 inhibitor LY in counteracting HFD, 8-week-old C57BL/6N male mice were challenged with a 60% HFD and orally treated with LY during 12 weeks. In parallel, mice maintained on either HFD or NC were used as reference groups (Figure 1A). HFD-fed mice receiving LY (HFD + LY) gained significantly less body weight (Figure 1, A and B) and accumulated lower amounts of s.c. adipose tissue (SAT) and visceral adipose tissue (VAT) than their counterparts receiving only the HFD (Figure 1C). To gain insight into the metabolic consequences of this treatment, we measured the concentrations of circulating glucose, TGs, and cholesterol at fasting. Also insulin tolerance tests (ITT) were performed in NC, HFD, and HFD + LY mice. Notably, the HFD + LY group exhibited decreased levels of circulating TG, with regard to HFD mice (Figure 1D). However, circulating cholesterol (*P* = 0.18), fasting glucose, and the ITT showed no major differences between obese animals under HFD and those treated with the drug (Figure 1, D and E). After sacrifice, H&E staining was performed in samples of epididymal adipose tissue (Figure 1F), and morphometric analysis revealed a rearrangement of adipocyte sizes in HFD-fed mice treated with LY (Figure 1G). Indeed, following the protocol from Parlee et al. (21), we found reduced adipocytes area and less hypertrophy in LY-treated mice than in the HFD group (Figure 1H). This matches diminished S6K1 function and the compromised adipogenic commitment of human BM-derived mesenchymal stem cells (BMSC) when induced to differentiate into adipocytes while challenged with LY (Supplemental Figure 1, A–C; supplemental material available online with this article; https://doi.org/10.1172/jci.insight.150461DS1). Also in liver samples, H&E staining revealed apparent changes in the lipid content (Figure 1I), as further shown by the amounts of TG and total cholesterol in tissue extracts, both being significantly depleted in HFD + LY to the levels found in NC-fed lean mice (Figure 1J). Our results indicate that LY protects against DIO and the deposition of fat in the liver, also ameliorating dyslipidemia in DIO mice.

### S6K1 blockage modifies adipose and liver gene expression programs.

**Figure 2:**
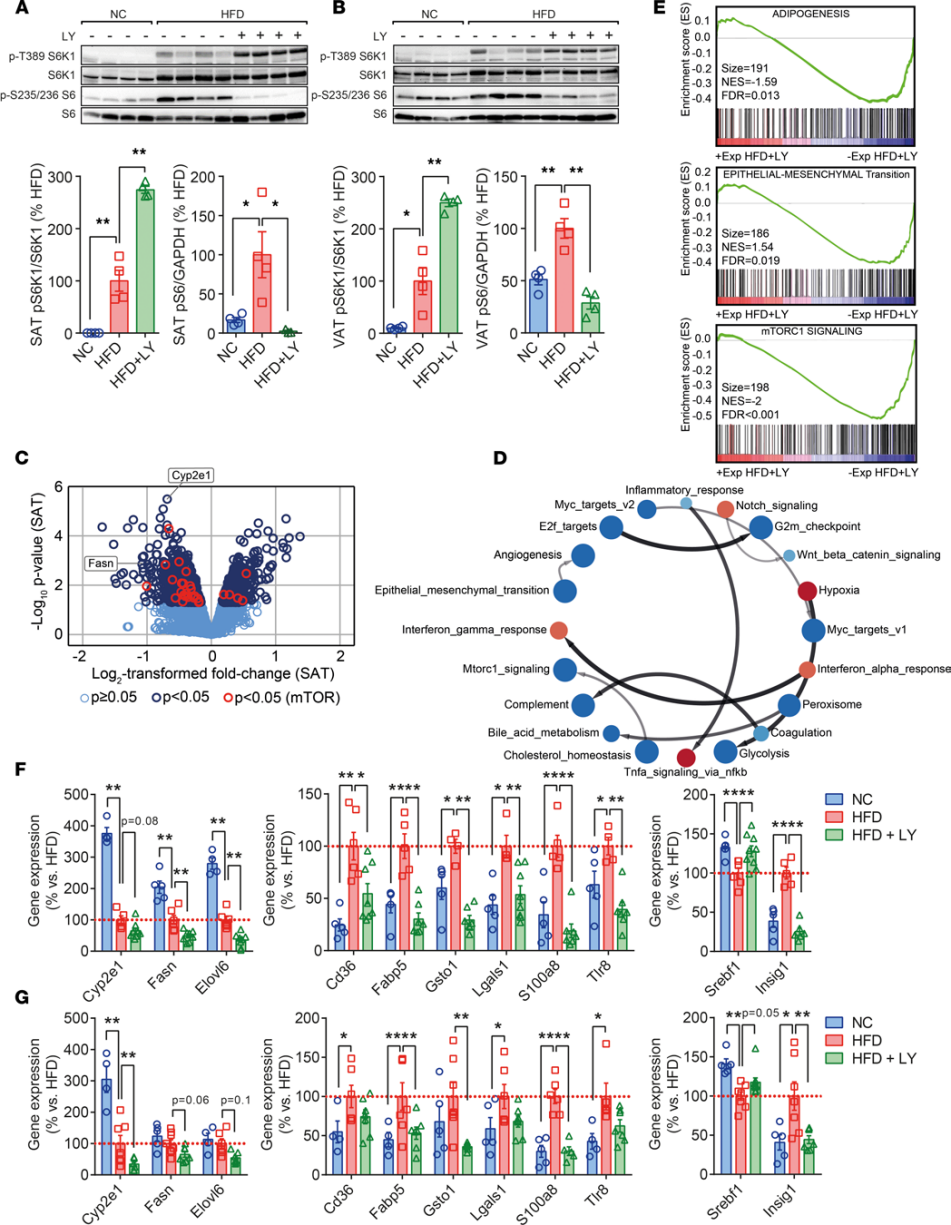
LY impacts white adipose tissue gene expression patterns. (**A** and **B**) Analysis of phosphorylated pS6 and pS6K1 in SAT (**A**) and VAT (**B**), and relative quantification as compared with high-fat diet–fed (HFD-fed) mice (100%). (**C** and **D**) Volcano plot and Gene Set Enrichment Analysis (GSEA) of transcripts modified in HFD + LY SAT when compared with HFD-fed mice (*n* = 3/group). Red and blue nodes represent positive and negative enrichment scores (FDR *P* < 0.05), respectively, and arrows show connections between signaling pathways with common genes in the leading edge subset. See also Supplemental Figure 2A. (**E**) Scheme illustrating the weight and enrichment score (ES) of variations in the expression (Exp) of genes related to the biological pathways most significantly downregulated in fat samples of HFD-fed mice when challenged with LY (HFD + LY). Red represents increased and blue represents decreased levels. (**F** and **G**) qPCR of gene candidates assessed in SAT (**F**) and VAT (**G**). Results from qPCR and Western blots are presented as mean ± SEM (*n* ≥ 4/group). Statistical significance was determined by the Dunnett’s multiple comparison procedure. Nontreated HFD-fed mice served as the reference group. *P < 0.05, **P < 0.01.

**Figure 3:**
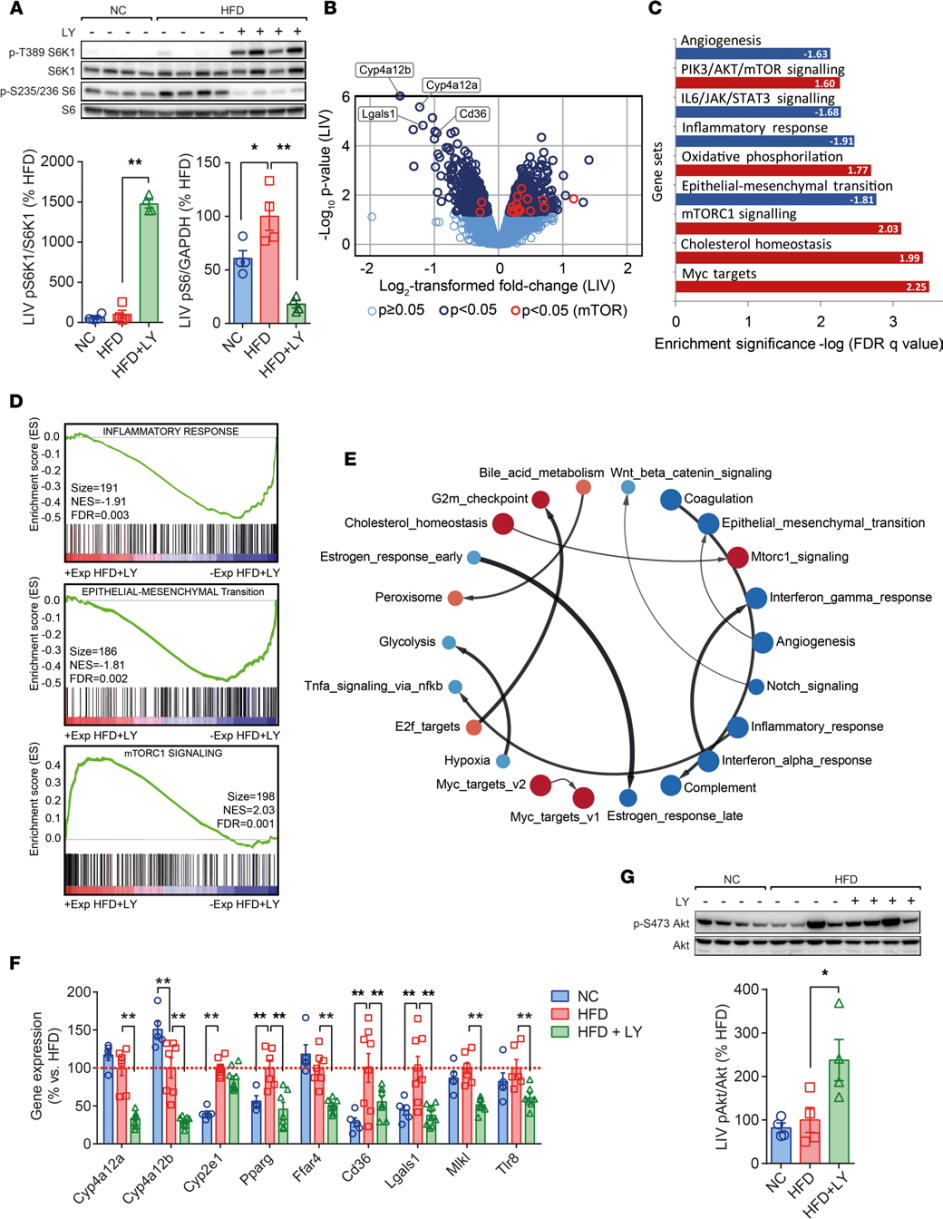
LY modifies liver function. (**A**) Analysis of phosphorylated pS6 and pS6K1 in the liver parenchyma of NC, HFD and HFD + LY mice. (**B**–**E**) The volcano plot shows variations in gene expression pattern of HFD + LY versus HFD mice (*n* = 3/group), while computational GSEA of such changes reveal the positive or negative impact in hallmark gene sets of biological relevance for liver performance, some of them being closely interrelated. (**F** and **G**) Validation of preselected target genes (qPCR), and analysis of the ratio between phosphorylated (p)Akt and total Akt in the liver of NC, HFD, and HFD + LY mice, with HFD mice being set as the reference group (%). Data are presented as mean ± SEM (*n* ≥ 4/group). Statistical significance was determined by the Dunnett’s multiple-comparison procedure. Nontreated HFD-fed mice served as the reference group. *P < 0.05, **P < 0.01.

S6K1 inhibition by LY was confirmed by Western blot in representative specimens of all groups (Figure 2, A and B, and Figure 3A, in adipose tissues and liver, respectively). Increased T389 pS6K1, due to the blockage of the phosphotransferase activity caused by the drug and reduced phosphorylation of the ribosomal protein S6 (^p-S235/236^S6), a downstream target of S6K1 (22), were analyzed as markers of impaired S6K1 activity in targeted tissues — i.e., SAT and VAT and in liver samples (LIV). We found that treatments with LY increased pS6K1 and decreased ^p-S235/236^S6 in treated animals, when compared with untreated HFD-fed mice, reaching a greater difference than with NC-fed mice. Then, the biological effects of this inhibition were studied by means of transcriptomic profiles assessed in SAT and LIV samples from HFD and HFD + LY mice. This provided a list of genes differentially expressed in adipose tissue (Figure 2C) and liver (Figure 3B) of HFD mice treated with the drug, when compared with HFD controls. Notably, gene set enrichment analysis (GSEA) and gene ontology (GO) term enrichment applied to expression patterns in SAT identified biological pathways that were widely attenuated (Figure 2D and Supplemental Figure 2A). Among them, adipogenesis, epithelial-mesenchymal transition (EMT), and mTORC1 signaling (Figure 2E) were the pathways most clearly challenged by treatment. Validation of microarray results by means of real time-PCR disclosed that genes related to the lipid biosynthesis (e.g., *Cyp2e1*, *Fasn*, *Elovl6*) were less expressed in SAT and VAT of HFD + LY mice, when compared with HFD-fed mice. A similar trend was seen in genes related to fatty acid uptake (*Cd36*, *Fabp5*), EMT (*Lgals1*), the inflammatory response (*Gsto1*, *S100a8*, *Tlr8*), and the control of fatty acids biosynthesis (*Srebf1*, *Insig1*), resembling the expression patterns revealed in NC lean mice (Figure 2, F and G). These results confirm the potential of reducing the adipogenic commitment and adipocyte hypertrophy in adipocyte progenitors (Supplemental Figure 1C), thus inhibiting the onset of obesity by a compound targeting S6K1 in adipose tissue.

In contrast, gene expression patterns in liver pointed at distinctive variations that included significant effects related to cholesterol homeostasis and mTORC1 signaling (increased), and the downregulation of transcripts associated with EMT, angiogenesis, and the inflammatory response of obese mice under treatment (Figure 3, C and D), among others (Figure 3E). Of note, expression of genes related to fatty acids biosynthesis (e.g., *Cyp4a12a, Cyp4a12b, Cyp2e1*, *Pparg*) and fatty acids uptake (*Ffar4*, *Cd36*) was compromised by LY, while the synthesis of mRNAs related to the EMT (*Lgals1*), and those representative of changes affecting the inflammatory response (*Mlkl*, *Tlr8*), was also downregulated (Figure 3F), mirroring — to some extent — what we found in fat samples. Intriguingly, an apparent overexpression of genes related to the mTORC1 signaling in HFD + LY mice was opposite to the results obtained in the adipose tissue of the same animals (Figure 3, B–D), suggesting the activation of different signaling cascades in fat and liver. Thus, we checked the activation of Akt, evaluated by its phosphorylation at S473 (hereafter indicated pAkt), as the expression of genes related to PIK3/AKT/mTOR was higher in HFD + LY livers. Consistently, pAkt was significantly upregulated in these livers (Figure 3G) but not in fat samples from HFD + LY mice (Supplemental Figure 2B), pointing at alternate mechanisms leading to distinctive variations accounting for physiological changes in adipose tissues and the liver parenchyma, the latter being more complex and relevant for lipid homeostasis.

Of note, treatments of LY on mouse hepatoma cells confirmed these in vivo results from liver samples. First, Hepa1-6 hepatocytes were prompted to accumulate lipid by a combination of palmitate-oleate (PA/OA), mimicking HFD-induced hepatosteatosis (23) and were treated with LY (PA/OA + LY) for 24 hours. Then, pS6K1, pS6, and pAkt; the amount of lipid (Oil Red O staining) and intracellular TGs; and the expression of preselected marker genes were quantified. We observed that LY blocked the kinase activity of S6K1 and, thus, diminished pS6 in these cells (Supplemental Figure 3A). In parallel, microscopic assessment of lipid droplets by Oil Red O staining (Supplemental Figure 3B), and quantification of TG by a colorimetric assay (Supplemental Figure 3C), revealed the significant reduction of these lipids in the LY-treated Hepa1-6 hepatoma cells. Also of interest, hepatocytes incubated with PA/OA and challenged with LY (PA/OA + LY) showed increased pAkt (Supplemental Figure 3A) and gene expression patterns coincident with those assessed in vivo (Figure 3F) — namely, decreased *Cyp4a12a/b*, *Lgals1*, *Mlkl*, and *Tlr8* (Supplemental Figure 3D). These data support the impact of LY measured in the liver transcriptome.

Another question of clear interest was whether treatments of LY modified the cellular composition and heterogeneity of adipose tissues (24), as analyses in S6k1^–/–^ mice showed important variations in this regard (13). Here, we utilized in silico deconvolution to computationally estimate the relative proportions of distinct cell types in bulk SAT and liver transcriptome data sets. Accordingly, the reanalysis of the transcriptomic profiles assessed in SAT depicted single variations accounting for changes in the extent of interstitial DCs (iDC) and epithelial cells (increased), and lower amounts of adipocytes (*P* = 0.073) in HFD-fed mice when treated with LY (Supplemental Figure 4A). In addition, a few significant changes were revealed in liver, as well — namely, the decreased expression of markers for proinflammatory type M1 macrophages and endothelial cells (Supplemental Figure 4B) in those specimens where LY dampens hepatic inflammation.

### Untargeted lipidomics in liver underscore the impact of LY on lipid handling.

**Figure 4:**
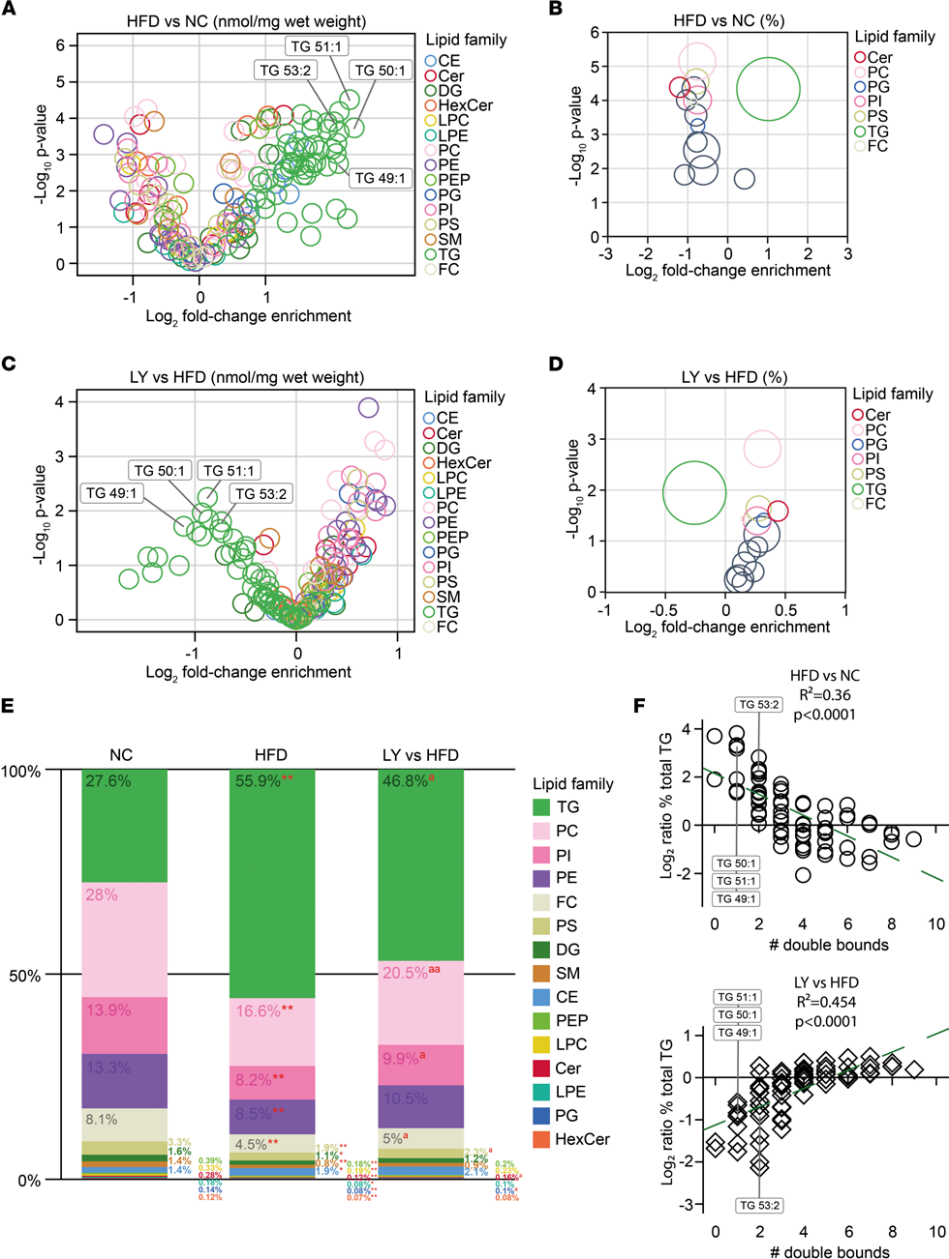
LY regulates triglycerides in the liver of diet-induced obese mice. (**A**–**D**) Volcano plots show variations in lipid species (left) and families (right panels) when comparing livers of HFD versus NC (**A** and **B**) and HFD + LY versus HFD mice (*n* = 4/group) (**C** and **D**). Right panels show colored circles for families with significant changes. (**E**) Stacked bars summarize relative amounts of lipid families in each experimental group and significant changes when comparing HFD versus NC (red asterisks), and HFD + LY versus HFD-fed mice (red superscript “a”). Statistical significance was determined by Fisher’s exact *t* test, and *P* values less than 0.05 (single) or 0.01 (double asterisk or superscript “a”) were considered significant. (**F**) Spearman’s test correlation analyses were applied to log_2_ ratios for each triglyceride (TG) species and the number of double bounds contained in their fatty acids, when comparing HFD versus NC (upper), and HFD + LY versus HFD (lower panel).

So far, our results demonstrated that oral administration of the S6K1 inhibitor LY in mice following a HFD regime (a) dampens fat expansion and hypertriglyceridemia, while (b) downregulating a number of molecular cascades in adipose — including adipogenesis, mTORC1 signaling, and inflammation — and (c) modulates the hepatic response to high amounts of fatty acids, such as the activation of key gene nodes related to PIK3/AKT/mTOR signaling and protection against steatosis. Next, we studied in all groups the hepatic lipidome by mass spectrometry. Our aim was to evaluate further therapeutic changes in the TG content and other lipid species related to fatty liver disease in HFD mice. Alterations in the lipid profile of HFD-fed mice when compared with NC (Figure 4, A and B), and in HFD + LY mice when compared with HFD-fed controls (Figure 4, C and D), are shown as volcano plots. Global variations in lipid families are also enclosed, and comparisons within the relative amounts of lipid families are represented in stacked bars (Figure 4E). Noteworthy, many TG species that were increased in samples from HFD when compared with NC mice (e.g., TG 49:1–3, 50:1–4, 51:1–2, 53:2, 54:2) were significantly reduced in the HFD + LY group (Figure 4, A and C). Indeed, significant reduction of the global amount of TG in HFD + LY mice was consistent with data obtained in serum (Figure 1D) and liver (Figure 1J), and also in Hepa1-6 cells (Supplemental Figure 3C). Strikingly, the main downregulation of hepatic TG accomplished by treatments of LY was quite heterogeneous and closely related (*R*^2^ = 0.45, *P* < 0.0001) to the number of double bonds contained in each TG species (Figure 4F). This means that LY mostly influenced the TG composed of saturated fatty acids (e.g., TG 49:1, 50:1, 51:1, 53:2), which are also the lipid species more prone to be upregulated by a sustained 60% HFD (*R*^2^ = 0.36, *P* < 0.0001) (Figure 4F). Also of interest, in HFD + LY mice, several species of phospholipids, including PC, PI, and PS, showed significant variations that were opposite to those reported in HFD, when compared with NC mice (Figure 4, A–D). Specifically, PC that appear more prevalent in NC-fed mice (28%) with regard to the mice under HFD (16.6%, *P* < 0.0001) are to certain extent maintained in HFD + LY mice (20.5%, *P* = 0.0016), highlighting preservation of this phospholipid with demonstrated efficacy in DIO and hyperlipidemia (25, 26). Noteworthy, changes in lipidomes — namely, those affecting TG (Supplemental Figure 5A) but also some variations in PC and PE (Supplemental Figure 5B) — were associated with a significant modulation in the expression of key genes related to the lipid metabolism network (e.g., increased *Enho*, *Zbtb16*, and *Slc2a1* and decreased *Cd36*, *Rgs16*, *Slc27a1*, and *Aebp1*), accomplished through the specific modification of hepatic S6K1 and variations in Akt signaling.

### S6K1 inhibition reduces s.c. fat mass and hepatosteatosis in obese mice.

**Figure 5:**
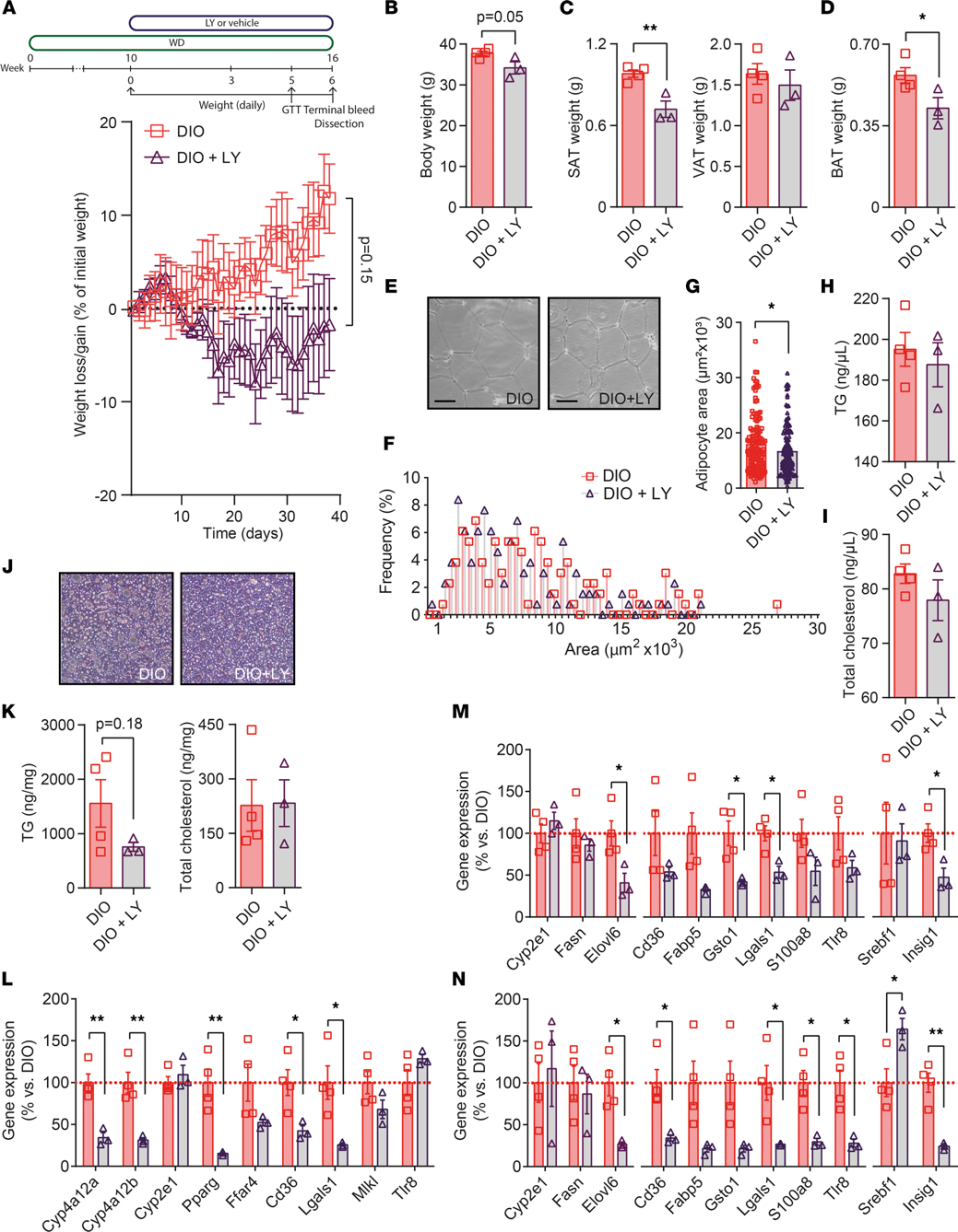
Impact of LY on obese mice. (**A**) Pipeline diagram for setting #2 and weight-loss curve in diet-induced obese (DIO) mice maintained on a high-fat and high-sucrose western diet (WD), or WD plus oral gavage of LY (DIO + LY). (**B**–**D**) Whole body weight and measures taken for depots of SAT and VAT, and brown adipose tissue (BAT) upon sacrifice confirm weight reduction. (**E**–**G**) H&E staining in SAT, distribution of adipocyte sizes (%), and total adipocyte area in DIO and DIO + LY mice. See also Supplemental Figure 7, C–E. (**H** and **I**) Circulating TG and cholesterol taken in 6-hour fasted mice upon sacrifice. (**J**) Representative 200× images of H&E staining in liver samples. (**K**) Quantification of the lipid content (TG and total cholesterol) in DIO and DIO + LY livers. (**L**–**N**) Real-time PCR of gene candidates from setting #1 were assessed in liver (**L**), SAT (**M**), and VAT samples (**N**). Data are presented as mean ± SEM. *n* = 4 for the DIO-control group, and *n* = 3 in the DIO + LY group. Statistical significance was determined by Fisher’s exact *t* test. *P < 0.05, **P < 0.01.

To assess whether drug-induced S6K1 inhibition does not simply modify transcriptomes and improve lipid homeostasis by suppressing exacerbated weight gain under a HFD, we studied the impact of LY on DIO mice. To set up this model, 8-week-old male C57BL/6N mice were fed a high-fat and high-sucrose diet (or Western diet), known to promote an obese phenotype (27) while fostering impaired metabolism and liver steatosis in C57BL/6 substrains (28). After 10 weeks, mice of ~35 g were treated with LY or vehicle during the following 6 weeks before being terminated (Figure 5A). In this setting, we analyzed the effects of LY on the liver, on the white fat, and in additional tissues, such as brown adipose tissue (BAT), muscle, pancreas, and brain. Samples from obese mice receiving LY (DIO + LY) confirmed the straight impact of the drug (i.e., high levels of pS6K1 plus diminished ^p-S235/236^S6 in treated animals) on liver, SAT, and VAT (Supplemental Figure 6, A–C). A less consistent blockage of S6K1 was observed in other tissues, leading, however, to an increased pS6K1 in BAT, muscle, and pancreas and reduced ^p-S235/236^S6 in the brain (Supplemental Figure 6, D–G). Furthermore, we did not observe consistently the indirect modulation of pAkt correlating with the impact of LY on the liver parenchyma and targeted depots of white fat (Supplemental Figure 6H). As in our first experimental setting of mice treated with LY during diet induction of obesity (Supplemental Figure 7A), treated obese animals consumed the same amount of food as the reference group (Supplemental Figure 7B) but showed a trend to a lessened final body weight (Figure 5B), mainly correlating with the lower amounts of SAT and BAT, as no changes were observed in the weight of depots of VAT (Figure 5, C and D). These results agree with the reduced size of adipocyte and less hypertrophy in SAT (but not in VAT) of LY-treated DIO mice (Figure 5, E–G, and Supplemental Figure 7, C–E). Here again, there was no signs of improved glucose tolerance (Supplemental Figure 7, F and G), and the nonsignificant but apparent downregulation of circulating TG and cholesterol in the DIO + LY group confirms, to some extent, the ability of S6K1 blockage in counteracting impaired lipid homeostasis (Figure 5, H and I). Also in concurrence with results obtained in our first experimental setting, H&E staining (Figure 5J) and colorimetric measures of the hepatic TG content (*P* = 0.18) in DIO + LY extracts (Figure 5K) pointed at changes affecting the build-up of fat in the liver parenchyma. Finally, gene expression measurements confirmed the impact of LY on some of the genes most clearly modified in our first experimental setting — namely, *Cyp4a12a, Cyp4a12b, Pparg*, *Cd36*, and *Lgals1* in the liver (Figure 5L) and *Elovl6*, *Cd36*, *Fabp5*, *Gsto1*, *Lgals1*, *S100a8*, and *Insig1* in SAT (Figure 5M) and/or VAT (Figure 5N). These results confirm that, beyond restraining fat mass expansion, LY may ameliorate dyslipidemia and the deposition of fat in the liver parenchyma by modifying adipose and liver performance in obese mice. Overall, the current pioneering preclinical findings reveal the potential of LY-induced S6K1 blockage as an efficacious therapeutic treatment to hamper fat mass expansion and relieve the burden of dyslipidemia and fatty liver in obese patients.

## Discussion

**Figure 6:**
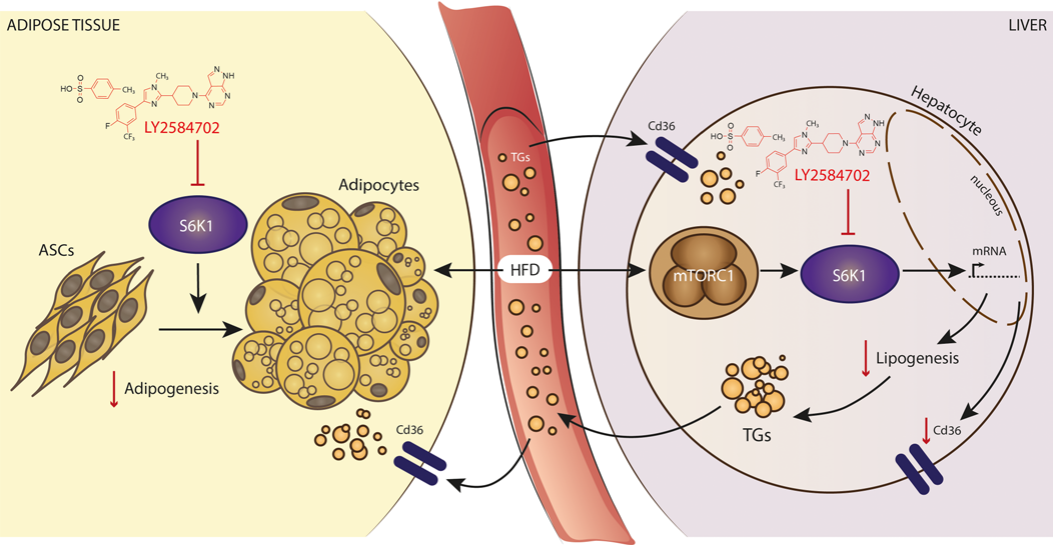
Study schematic. The specific blockage of S6K1 activity modifies gene expression patterns relevant for adipogenesis, adipose tissue accretion, and liver function. These changes combined align with reduced adipocyte size and improved lipididomic signatures in the liver, while hypertriglyceridemia and hepatosteatosis were prevented to some extent in diet-induced obese mice using the LY2584702 tosylate, a compound directed against S6K1 for clinical development.

The worldwide epidemic of obesity is the chief cause of the dramatic increase in a number of pathologies (29–31). Because of dominant genetic traits that encourage caloric intake, food availability, the scale of the epidemic, and the explosion of medical costs related to the burden of metabolic diseases, there is a critical need to develop novel strategies to treat obesity and its sequelae. Inhibiting the recruitment of adipose-derived stromal cells to adipose depots has been considered previously as a potential approach for therapeutic interventions against obesity (32, 33). However, developing this opportunity will require the understanding of the many molecular components that may regulate this recruitment, and it will require the identification of novel inhibitors safe and useful in such intervention therapies. The present study outlines the impact of LY, a ribosomal protein S6K1 inhibitor with demonstrated efficacy in glioblastoma and colon carcinoma xenograft models (19), as a therapeutic compound to block the onset of DIO and related disturbances by regulating the bulk of adipose tissue and accumulation of fat in the liver parenchyma. As first hypothesized, treatments with LY in DIO mice compromised the commitment of fat cell progenitors within depots of white adipose, as shown by gross physiological and histological analysis, and by transcriptional variations assessed in fat samples. Besides the impact on fat mass, the therapeutic blockage of hepatic S6K1 also ameliorated steatosis and dyslipidemia through the molecular changes implemented in liver, as depicted by transcriptomics and lipid signatures. Notably, the drug performed acceptably well also in preestablished obese mice — decoupling, to some extent, the impact on gene expression and improved lipid homeostasis from reduced weight gain during the course of a sustained HFD regime. Thus, our findings indicate that LY may represent an efficacious therapeutic agent to relieve not only the burden of hyperplastic fat depots affecting metabolism, but also the progression of hypertriglyceridemia and fatty liver disease in obese patients (Figure 6).

S6K1 is a key factor involved in the recruitment of precursor fat cells to the adipogenic lineage (9). Mice lacking this effector are protected against DIO due to a lesion in adipogenesis that limits fat mass expansion (13, 17). Concomitant to reduced amounts of adipose tissue, this deficiency causes enhanced osteogenesis and protects against age-related pathologies, leading to an increased lifespan (34). In the present study, we wanted to investigate the therapeutic blockage of S6K1, mimicking the protection against obesity shown by animals lacking this effector. We studied in detail the metabolic and molecular effects depicted in SAT and VAT, as well as in the liver, since they are the major metabolic tissues related to lipid homeostasis. Accordingly, mice receiving a HFD and treated with the drug showed a significant reduction of body weight and accumulated less white adipose tissue (WAT), which is in line with the phenotype of our S6K1-deficient mice (13). Also in agreement with our previous mouse model, morphometric analysis of the adipose tissue showed that fat cells from animals treated with LY present less adipocyte hypertrophy than nontreated controls, and the ability of this compound to block the commitment of precursor stem cells to the adipocyte phenotype was confirmed in vitro. Finally, microarray results and real-time PCR revealed the impact of LY on genes related to fatty acid biosynthesis and lipid uptake, which were expressed to a lesser extent within HFD + LY adipose tissues. For instance, *Srebf1* — a key mediator in the regulation of fatty acid anabolism (35), also triggered by mTORC1 (36, 37) — showed lower expression levels in the HFD group, when compared with NC and HFD + LY mice. This transcription factor is known to be regulated by insulin-induced gene protein 1 (INSIG1), which retains the SCAP/SREBP complex in the ER (38), thus blocking the functional activation of SREBP. Accordingly, *Insig1* is significantly increased in obese rodents (39, 40), while it deters differentiation and lipogenesis in cultured adipocyte precursor cells (40). In the current study, drastic decrease of *Insig1* was observed in the SAT and VAT of HFD + LY mice as compared with the HFD group, similar to the expression pattern for genes related to fatty acid intake, EMT, and inflammation (Figure 2, F and G). In fact, only the expression of genes directly related to proper adipocyte performance — namely, *Cyp2e1*, *Fasn*, and *Elovl6*, among others (41) — was decreased not only in HFD-fed mice, but also in obese animals; it was further compromised by LY in our models, pointing to the impaired adipocyte commitment referred above.

Since hepatic depletion of S6K1 protects against diet-induced hepatosteatosis (18), we also examined changes in liver morphology, gene expression patterns, and lipid composition responding to the blockage of S6K1. Coincident with an alleviation of hepatic steatosis, we computationally detected the downregulation of IL-6/JAK/STAT3 signaling and the inflammatory response (e.g., *Tlr8, Mlkl*), together with impaired EMT (*Lgals1*) in the liver, while the expression of genes related to mTOR was enhanced by LY (Figure 3B). The PIK3/AKT/mTOR signaling pathway and cholesterol homeostasis are closely interrelated (42). PIK3/AKT, when triggered by insulin, promotes the activation of mTORC1 (43), which in turn mediates the pS6K1 (14). Probably, under conditions of hyperphosphorylated and dysfunctional S6K1 upon treatment with LY, the molecules of phosphorus remain upstream of the cascade, favoring the overactivation of PIK3/AKT and mTORC1, as shown in HFD + LY livers and Hepa1-6 cells. The mechanisms resulting in the different transcriptomic regulations in fat and liver led by LY remain a key question we seek to answer in our future studies.

Serum TG were clearly diminished in obese mice when treated with the drug, as well as TG quantified in the liver parenchyma and in cultured mouse hepatocytes. This may suggest that mice treated with LY oxidize hepatic TG faster than nontreated mice, as β-oxidation is enhanced in both *S6k1*-deficient mice (17) and the liver-specific *S6k1-*depleted mice (18). However, the current available data do not allow proper evaluation of the energy expenditure component, which can be altered over time by the treatment, and deserves further attention in future investigations. Also of interest, PC, PI, and PS were proportionally increased in the liver of the HFD + LY group, in detriment of monosaturated TG, resembling to some extent the lipid signature of NC mice (Figure 4C). Natural phospholipids are available as a heterogeneous mixture of PC with different fatty acyl chains. Previously, a study comparing hepatic lipidomes of HFD-induced obese and metabolically compromised mice to NC-fed lean animals showed significant differences in TG and PC species (44), matching our current results. Ether phospholipids have antioxidant properties and have been found decreased in serum of obese patients (45), while PI appear to show antiobesity effects (46), also correcting dyslipidemia to some extent (47). These findings together support a positive balance of lipid species that may help to maintain the performance of liver even through conditions of HFD, also ameliorating obesity-related hepatosteatosis and dyslipidemia.

Numerous studies have pointed the relevance of p70S6K in conveying signaling cascades related to the burden of obesity and metabolic disturbances (48). Thus, the potential utility of inhibitors directed against S6K1 — namely, PF-4708671 (49) (developed by Pfizer) and LY2584702 (19) (developed by Lilly) — has raised as a feasible therapeutic option in the field (20). Here, we have studied the functional and mechanistic impact that orally administrated LY exerts on adipose tissues and liver, counteracting the effects of a very high–fat dietary regime. We conclude that the use of this specific p70S6K inhibitor hampers the onset of obesity and associated disturbances, such as the progression of fatty liver and hypertriglyceridemia, even though insulin sensitivity did not improve in our treated mice. This may be due to the decreased expression of Insulin receptor substrate 1 (IRS-1), the main target protein of mTORC1-S6K1 signaling (7), in mice under a dietary regime very rich in fatty acids (50). The phenomenon could also prevent the inhibitory feedback that pS6K1 exerts on insulin-induced glucose uptake through promoting inhibitory phosphorylation of multiple serine residues in IRS-1 (51), thus maintaining some degree of insulin resistance even when S6K1 is inhibited. Altogether, these pioneering findings shed light on the molecular mechanisms related to the therapeutic impact of compounds directed against S6K1 on the occurrence and development of DIO and related diseases. They warrant further investigation targeting the potential of LY2584702 as an efficacious therapeutic agent to relieve the burden of dyslipidemia and fatty liver disease in obese patients.

## Methods

### In vivo settings.

Eight-week-old male C57BL/6N mice from Charles River Laboratories were housed in a room with controlled temperature and a 12:12 light/dark cycle (6 a.m.–6 p.m.). Drinking water was provided ad libitum and consisted of running water through a reverse osmosis filter and chlorinated to 9–13 ppm. Bottles were changed weekly with autoclaved bottle and cap. In our initial approach (setting #1), mice were fed with either a standard laboratory chow (12.1 kJ/g; 4% fat, 48% carbohydrate, and 14% protein; Diet 2014S, Teklad Global Diets; Harlan) or a HFD (60% kcal from fat, 20% from protein, 20% from carbohydrate; Research Diets Inc.). During 3 months, mice were treated every 12 hours by oral gavage (25 mg/kg) with the S6K1 inhibitor LY (Axon Medchem BV) or vehicle (30% PEG300 [Sigma-Aldrich], 5% Tween80 [Sigma-Aldrich], and 65% water). Previous assessment of drug dosage and activity was performed during experiment set up (Supplemental Figure 8, A–D), and following Madala et al. (52). After the first 2 weeks of treatment, treated and control mice were challenged with the HFD or maintained under NC (only controls) for 10 weeks. To rule out changes merely related to a lessened weight gain, DIO mice were also treated with LY or vehicle (setting #2). Here, 8-week-old male C57BL/6N mice were fed with a high-fat and high-sucrose Western diet (45% kcal from fat, 15% from protein, 41% from carbohydrate; TD.08811, Harlan Laboratories) for 4 months and challenged with LY or vehicle during the last 6 weeks. The treatment was initiated when these mice weighed 34.3 ± 2.1 g. In both settings, mice growth and body weight were recorded daily, and parameters of food intake and blood glucose were measured. The metabolic phenotype was assessed between weeks 11–15 and included i.p. ITT (in setting #1) or oral glucose tolerance tests (performed in setting #2), as well as blood chemistry (determination of glucose, cholesterol, and TGs in serum and liver necropsies from both settings). At week 12–16, mice were terminated by cardiac puncture and epididymal and inguinal WAT, and liver samples for each mouse were embedded in paraffin or snap frozen in liquid N_2_ and stored at –80ºC. Besides liver and WAT, analyses of different tissues were performed in setting #2 to disclose the potential impact of LY in the BAT, muscle, pancreas, and brain.

### ITT and GTT.

ITT was performed in 18-week-old animals from setting #1 fasted for 6 hours. Blood glucose was measured at 0, 15, 30, 45, and 60 minutes from tail tip with an Accu-Check Active glucometer (Roche) after an i.p. injection of 0.75 U/kg insulin (Humulin R U-100, Lilly). GTT was performed in obese mice from setting #2, receiving LY or vehicle during 5 weeks, and fasted for 12 hours before the assay. There, blood glucose was measured at 0, 15, 30, 60, and 120 minutes after an i.p. injection of a 20% glucose solution (Glucocemin 33%, B. Braun). The areas under the glucose curves were evaluated according to the trapezoidal rule in each animal.

### Histological analysis.

Fat pads and liver pieces of mice from each group were fixed in formalin and embedded in paraffin; they were then cut in 5 μm sections and stained with H&E. ImageJ software (NIH) was used within 20× images to analyze the area of 140–180 adipocytes. Cells were outlined by enhance contrast function, and the calculated area was obtained for each as previously described (21).

### Biochemistry.

Cholesterol and TGs were studied in serum and liver necropsies from both settings. In total, 30 mg of frozen livers were homogenized with a combination of chloroform/isopropanol/IGEPAL CA-630 (7:11:0.1) or 5% IGEPAL CA-360 (I8896, Sigma-Aldrich). These lipids were measured in homogenates and serum using the Triglyceride (MAK266) and Cholesterol (MAK043) Quantification Kits (Sigma-Aldrich).

### In vitro models.

Hepa1-6 (ATCC CRL-1830) murine hepatoma cells were cultured in DMEM supplemented with 10% FBS (Thermo Fisher Scientific), 1% penicillin and streptomycin, and 1% HEPES and NaPyr at 37°C and 5% CO_2_. A combination of PA (200 μM) and OA (300 μM) (Sigma-Aldrich) was used to induce steatosis in these hepatocytes, as previously described (23, 53). Then, cells challenged with PA/OA were also treated with 1 μM LY or vehicle. Treatments were performed 24 hours after seeding and were maintained during 24 hours before harvesting. Human BMSC were cultured and induced to differentiate into mature adipocytes with commercially available differentiation media (ZenBio Inc.). During differentiation, cell media were supplemented with 1 μM LY or vehicle. Nondifferentiating BMSC were maintained in BMSC growth medium supplemented with vehicle.

### Oil Red O staining.

Oil Red O staining was used to determine intracellular lipid content in cells. Cultures of adipocytes and hepatocytes were washed with PBS and fixed with paraformaldehyde 7% (Sigma-Aldrich) for 1 hour. Then, cells were dipped in isopropanol 60% before being completely dried and stained with filtered 5% Oil Red O in isopropanol for 10 minutes at room temperature; they were then washed up to 4 times with distilled water. Pictures were taken using an inverted microscope Leica DM IL LED. After they were completely dried, cells were dipped in 100% isopropanol and incubated up to 10 minutes to elute Oil Red O. Optical density was measured at 500 nm using a PowerWave XS spectrophotometer (BioTek Instruments).

### Gene expression.

Total RNA was extracted from mouse tissues and cells using RNeasy Mini Kit (Qiagen), and concentrations were measured using a Nanodrop ND-1000 Spectrophotometer (Thermo Fisher Scientific). Total RNA was reverse transcribed to cDNA using High Capacity cDNA Archive Kit (Applied Biosystems). Commercially available TaqMan primer/probe sets (Thermo Fisher Scientific) and forward/reverse SYBR Green paired primers were used to analyze the gene expression with a Light Cycler 480 II (Roche Diagnostics). The peptidyl propyl isomerase A (Ppia) and β-actin (Actb) were used as housekeeping references. TaqMan assays and SYBR Green forward/reverse primer sets are listed as Supplemental Table 1.

### Western blot.

Cells and tissues were homogenized using a T10 basic ULTRA-TURRAX (IKA-Werke GmbH). Protein was extracted using CHAPS lysis buffer, containing 0.3% CHAPS, 40 mM HEPES, 120 mM NaCl, 1 mM EDTA, 10 mM pyrophosphate, 15 mM sodium orthovanadate, 50 mM sodium fluoride (NAF), and 10 mM glycerol phosphate and supplemented with protease and phosphatase inhibitors. Cells were washed with cold PBS and collected by scraping on ice with cell lysis buffer (Cell Signaling Technology). Cell lysates were kept in agitation for 30 minutes. Tissue and cell lysates were centrifuged at 10,000*g* for 10 minutes at 4°C to discard cell and tissue debris. Protein was quantified using the Pierce BCA Protein Assay Kit (Thermo Fisher Scientific). Protein extracts were separated using 4%–20% precast polyacrylamide gel (Bio-Rad) and transferred to polyvinylidene fluoride (PVDF) membranes (GE Healthcare). Membranes were blocked with 5% BSA (PanReac AppliChem) in 0.1% TBS-Tween20 (Sigma-Aldrich). Membranes were incubated with rabbit anti–^p-S235/236^S6 (catalog 2211), anti–^p-T389^S6K1 (catalog 9205; referred to as pS6K1), anti-S6K1 (catalog 9202), anti-GAPDH (catalog 2118), anti–^p-S473^Akt (catalog 9271; referred to as pAkt), and anti-Akt (catalog 9272), followed by incubation with a Polyclonal Swine Anti-Rabbit Immunoglobulin/HRP (catalog P0399, Agilent Technologies). All primary antibodies were purchased from Cell Signaling Technology and prepared at 1/1000 in 5% BSA in 0.1% TBS-Tween20. Protein signal was detected by chemiluminescence with an iBright CL1000 Imaging System (Thermo Fisher Scientific). Unedited gels are available in the supplemental material.

### Transcriptomes.

Microarray gene expression profiles were obtained in samples of s.c. inguinal adipose tissue and liver samples from 3 control HFD and 3 HFD-fed mice treated with LY (HFD + LY). Purified RNA was processed according to the following: GeneChip WT PLUS Reagent kit (P/N 703174 2017) and Expression Wash, Stain and Scan User Manual (P/N 702731 2017) (Affymetrix Inc). RNA integrity was checked with Agilent 2100 Bioanalyzer (Agilent Technologies). For statistical analysis, R programming environment (R Core Team [2018] version 3.4.4) was used together with different Comprehensive R Archive Network and Bioconductor packages (54, 55). Complete gene expression profiles have been deposited in the community-endorsed repository Gene Expression Omnibus (GEO, http://www.ncbi.nlm.nih.gov/geo/, accession no. GSE193028) following MIAME compliant guidelines. The analysis of lists of genes showing significant changes when compared with control was performed with GSEA (https://www.gsea-msigdb.org/gsea/index.jsp) and based on the hallmark GO set (56, 57). Pathway diagrams were also generated using Enrichment Map application in Cytoscape (v.3.7.2) (58) and the Hallmark collection.

### Lipidomes.

The analysis of lipids was performed by direct flow injection analysis (FIA) using either a triple quadrupole mass spectrometer (FIA-MS/MS; QQQ triple quadrupole) or a hybrid quadrupole-Orbitrap mass spectrometer (FIA-FTMS; high-mass resolution). FIA-MS/MS (QQQ) was performed in positive ion mode using the analytical setup and strategy described previously (59–61). Lipidomic data have been made publicly available in Figshare (dx.doi.org/10.6084/m9.figshare.18393854).

### Statistics.

Data are presented as mean ± SEM. Sample sizes (*n*) are mentioned on each figure legend. Two-way ANOVA followed by the Šidák’s correction and adjusted *P* values for repeated measures was used to counteract the problem of multiple observations made sequentially in time, as in weight gain assessments. Dunnett’s test was applied on other measures of the first experimental approach, with 3 groups of animals, one of which served as a reference group (i.e., nontreated HFD-fed mice). Statistical significance was determined by 2-tailed Fisher’s exact *t* test in comparisons between 2 groups. Familywise significance and confidence levels were set at 0.05 (95% CI). Statistics and plots were performed with GraphPad Prism v8.0 (GraphPad Software) and IBM SPSS Statistics 23 (IBM Analytics).

### Study approvals.

The present studies in animals (protocol no. 201617-10 for setting #1, and protocol no. LABAE19004LLOB for setting #2) were reviewed and approved by the Animal Experimentation Ethics Committee of the Bellvitge Biomedical Research Institute (IDIBELL), and they were carried out in accordance with the National Board of Climate Action, Food and Rural Agenda of the Generalitat de Catalunya.

## Author contributions

AL and FJO conducted research, analyzed data, performed statistical analysis, and wrote the manuscript. SRV, JL, JMMN, and NB conducted research (hands-on conduct of the experiments and data collection). NB, DLN, and RRB analyzed data and performed statistical analysis. VDN, YZ, MH, GL, and VMO carried out lipidome analyses and provided important intellectual content. GT, RRB, JMFR, SCK, and FJO designed the research, developed project conception, and revised the manuscript. FJO contributed most to this study and, therefore, is listed last.

## Supplementary Material

Supplemental data

## Version 1 06/23/2022

In-Press Preview

## Version 2 07/22/2022

Electronic publication
