# Supplementary material for: A compound directed against S6K1 hampers fat mass expansion and mitigates diet-induced hepatosteatosis
Source: JCI Insight. 2022 Jul 22;7(14):e150461. doi: 10.1172/jci.insight.150461 (PMC9431684; doi:10.1172/jci.insight.150461)

SUPPLEMENTAL FIGURE LEGENDS

Supplemental Figure S1. LY2584702 tosylate deters adipogenesis in human bone marrow stem cells (BMSCs): **(a)** Phosphorylated (p)S6K1, S6K1, pS6, and Gapdh (housekeeping) levels (WB), and relative quantification, taking “mature” adipocytes (mAd) as the reference group (%). BMSCs stands for undifferentiated bone marrow stem cells (n=2 biological replicates/group). **(b)** Representative Oil O-Red staining images and quantification of the lipid content of BMSCs when maintained under non-differentiating conditions, and when prompted to differentiate into mature adipocytes with (mAd+LY) or without (mAd) 1µM LY added to the differentiation media (n=4/treatment). **(c)** Gene expression levels of genes related to adipogenesis and adipocyte performance. Data is presented as mean ± S.E.M. Statistical significance was determined by the Dunnett’s multiple comparison procedure, and differentiated mAd cells were taken as the reference group. *p<0.05, **p<0.01.

Supplemental Figure S2. **(a)** Gene Set Enrichment Analysis (GSEA) of genes modified in subcutaneous adipose tissue (SAT) of HFD+LY *versus* HFD mice showed the overall downregulation of multiple hallmarks, including but not limited to mTORC1 signalling, EMT and adipogenesis. **(b)** Western blot analysis of phosphorylated (p)Akt and total Akt in SAT and visceral (VAT) adipose tissue indicated no significant differences between HFD+LY and HFD mice (n=4/group). Data is presented as mean ± S.E.M. Statistical significance was determined by the Dunnett’s multiple comparison procedure. Non-treated HFD-fed mice served as the reference group. *p<0.05.

Supplemental Figure S3. In hepatoma Hepa1-6 challenged with a combination of palmitate/oleate (PA/OA) to induce steatosis, 1µM LY2584702 **(a)** compromised S6K1 function, **(b)** reducing the size and/or number of lipid vacuoles contained in the cytoplasm (Oil-Red O staining), **(c)** leading to significant changes in the triglyceride (TG) content, and **(d)** modifying measures of gene expression assessed for target genes that mirrored to some extent the impact of the drug in HFD and DIO mice receiving LY (see in **Figures 3f** and **5l**). Data is presented as mean ± S.E.M. Statistical significance was determined by the Dunnett’s multiple comparison procedure, taking PA/OA hepatocytes as the reference group. *p<0.05, **p<0.01.

Supplemental Figure S4. Analysis of cell subset and deconvolution of microarray results for **(a)** subcutaneous adipose tissue (SAT) and **(b)** liver. Boxplots show average xCell scores for enriched cell types in the HFD reference group (red) and HFD+LY treated mice (blue plots). Statistical significance was determined by Fisher’s exact t-test. Only P values less than 0.1 are shown.

Supplemental Figure S5. **(a)** Triglycerides degradation pathway in relation to differentially expressed genes, and **(b)** interactive network of functional associations within genes and lipids differentially expressed in liver of HFD+LY and HFD mice,

using the Core Analysis function and the Ingenuity Pathways Knowledge base included in the Ingenuity Pathway Analysis (IPA) software. Colour indicates the activation states: “red”: upregulated; “green”: downregulated; “grey”: undetermined; “white”: molecules that are related to this pathway/network (based on the IPA knowledge base) but are not differentially expressed in the current set of results.

Supplemental Figure S6. Phosphorylated (p)S6K1, S6K1, pS6, and Gapdh (housekeeping) in **(a)** SAT, **(b)** VAT, **(c)** liver, **(d)** BAT, **(e)** muscle, **(f)** pancreas, and **(g)** brain necropsies from diet-induced obese (DIO) mice challenged with LY (n=3) or vehicle (n=4). **(h)** Phosphorylated (p)Akt vs total Akt was also measured in SAT, VAT and liver samples. Data is presented as mean ± S.E.M. Statistical significance was determined by Fisher’s exact t-test. *p<0.05, **p<0.01.

Supplemental Figure S7. Food consumption in our **(a)** setting #1 and **(b)** setting #2 depicts no significant variations in mice receiving LY. **(c)** H&E staining in VAT, **(d)** distribution of adipocyte sizes (%), and **(e)** total adipocyte area in DIO and DIO+LY mice (see results for SAT in **Figures 5e-g**). No significant changes in **(f)** measures of fasting glucose nor **(g)** the area under the curve (AUC) of glucose tolerance tests (GTT) were found, yet the DIO+LY group showed a trend over time that may suggest a faster recovering of glucose levels. Statistical significance was determined by Fisher’s exact t-test. *p<0.05.

Supplemental Figure S8. Trials and experimental set up: **(a)** Assessment of the impact of two dosages (i.e., 25 and 50 mg/kg) of orally administrated LY2584702 tosylate on subcutaneous fat samples from 2 and 6 month-old male mice. Phosphorylated (p)S6 and total S6K1, normalized against GAPDH, show no significant differences related to age nor to the amount of drug provided by oral gavage. **(b)** Timespan assessment of drug-related S6K1 inhibition in adipose and liver indicated ≤12h-lasting effects in adipose and liver. **(c)** Impact of the drug on mice under different dietary conditions showed the strongest effect in the adipose of HFD-fed mice, **(d)** while this diet did not seem to alter the activation of S6K1 in liver samples.

SUPPLEMENTAL TABLE

**SYBR Green**	**Forward primers**	**Reverse primers**
Cd36	CATTTGCAGGTCTATCTACG	CAATGTCTAGCACACCATAAG
Cyp4a12a	GTTCTTTACCCAAAGGTATCC	AGAAGGATCAAACACCTCTG
Cyp4a12b	ATGCCCTATACTACCATGTG	GATCAAACACCTCTGGATTTG
Elovl6	GTTTATTAATCCTCCCGCTG	CTTCTCTGGAAGTGTTTTCC
Fabp5	ATGAAAGAGCTAGGAGTAGG	TACAAGAGAACACAGTCGTC
Gsto1	CAAGCTTTGTTAGGTCGAAG	CTCTTGTAATTATCCATGCCC
Insig1	CTAGTGCTCTTCTCATTTGG	GATACAGTAAACCGACAACAG
Lgals1	CAGGTCTCAGGAATCTCTTC	CAGGTTTGAGATTCAGGTTG
Mlkl	GTTCAACGATATATGTCTCCC	AGAATCACAGCCTTCAAATG
S100a8	ATACAAGGAAATCACCATGC	ATATTCTGCACAAACTGAGG
Tlr8	TAGAACATGGAAAACATGCC	ATACTTGCCTATAGTTTGGGG
Actb	GATGTATGAAGGCTTTGGTC	TGTGCACTTTTATTGGTCTC
ELOVL6	AGTATATTCGGTGCTCTTCG	TTAGCACAAATGCATAAGCC
PPIA	ATGGTTCCCAGTTTTTCATC	CTCCACAATATTCATGCCTTC

**TaqMan assays**	**Reference #**
Cyp2e1	Mm00491127_m1
Fasn	Mm00662319_m1
Ffar4	Mm00725193_m1
Pparg	Mm00440940_m1
Srebf1	Mm00550338_m1
Ppia	Mm02342430_g1
ACACA	Hs01046047_m1
ACLY	Hs00982738_m1
CD36	Hs00354519_m1
FASN	Hs01005622_m1
LEP	Hs00174877_m1
PPARG	Hs01115513_m1
SCD	Hs01682761_m1
SLC2A4	Hs00168966_m1
SREBF1	Hs01088691_m1
THRSP	Hs00930058_m1
PPIA	Hs04194521_s1

SUPPLEMENTAL FIGURE S1

a

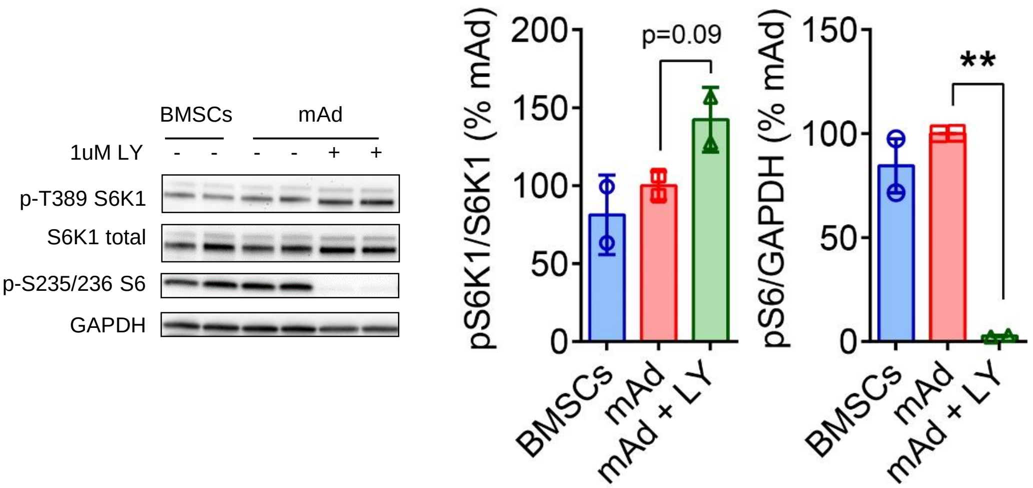

b

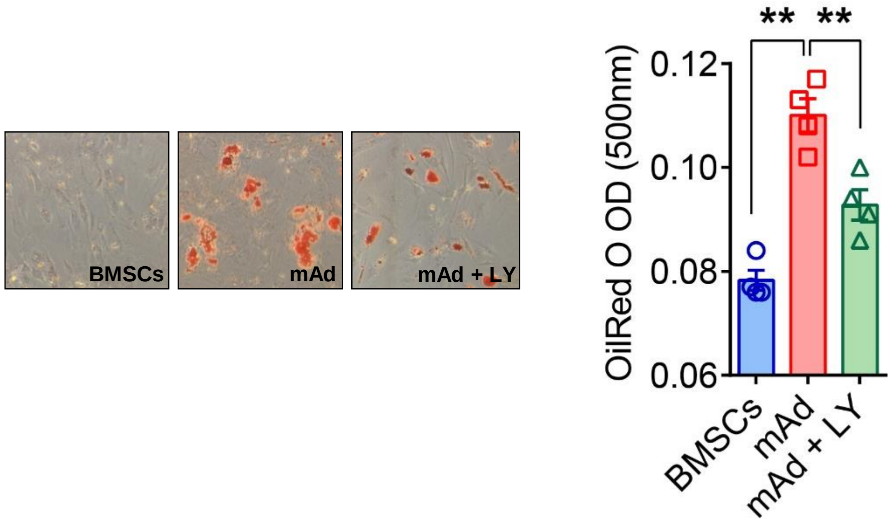

c

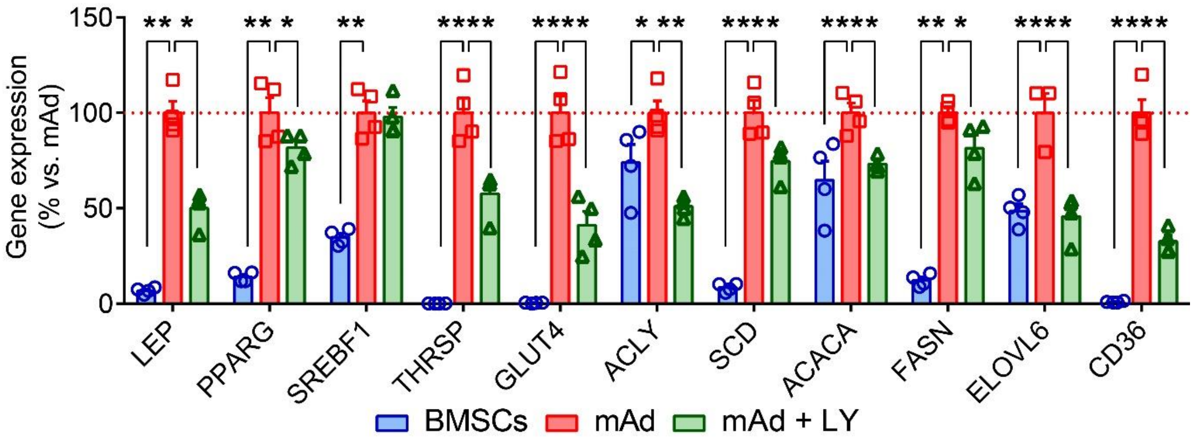

SUPPLEMENTAL FIGURE S2

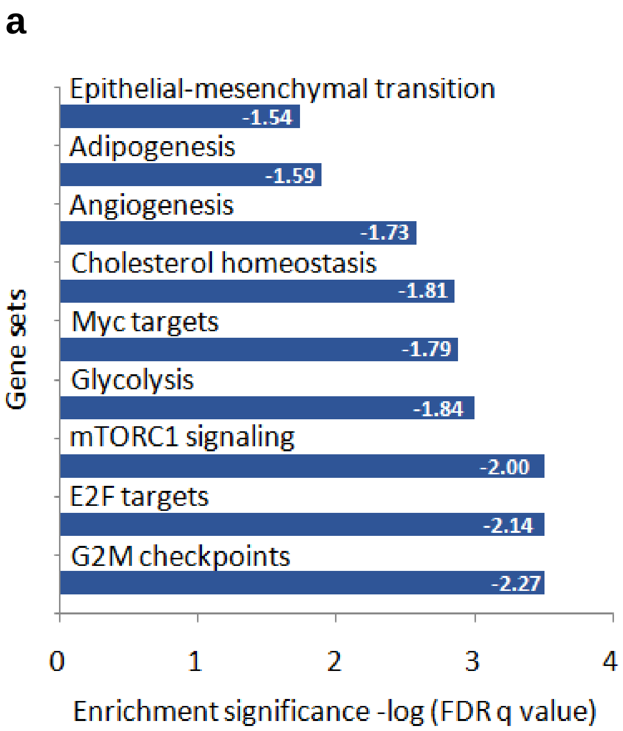

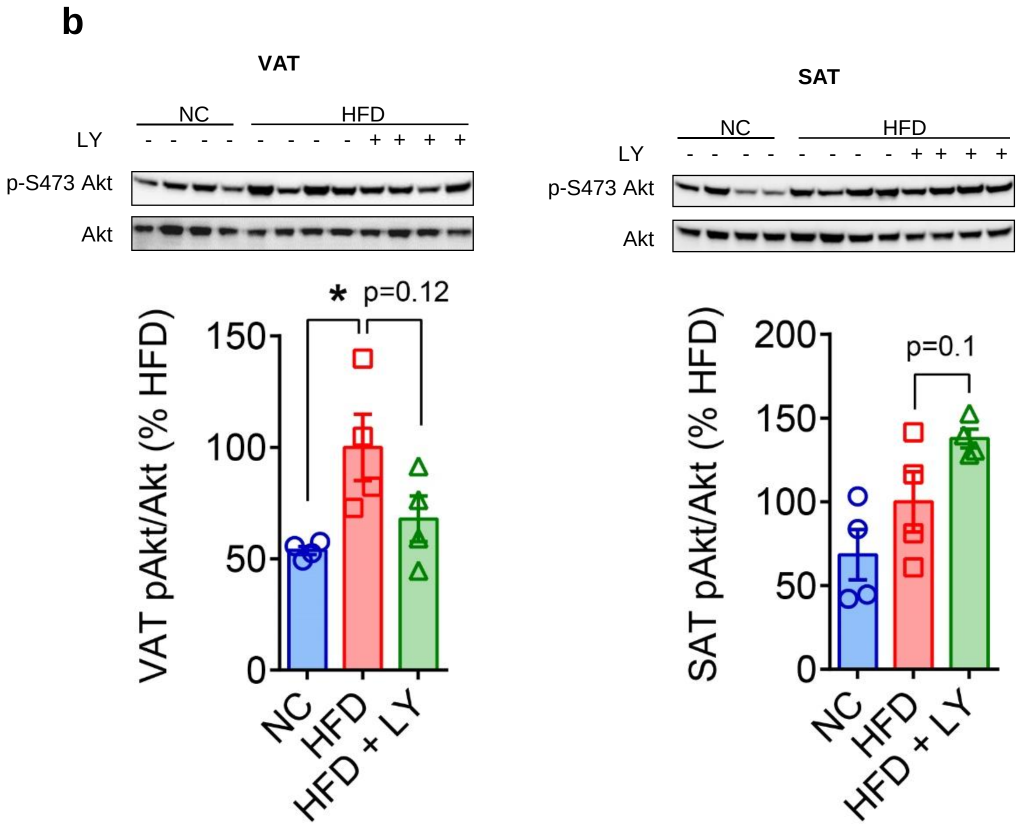

SUPPLEMENTAL FIGURE S3

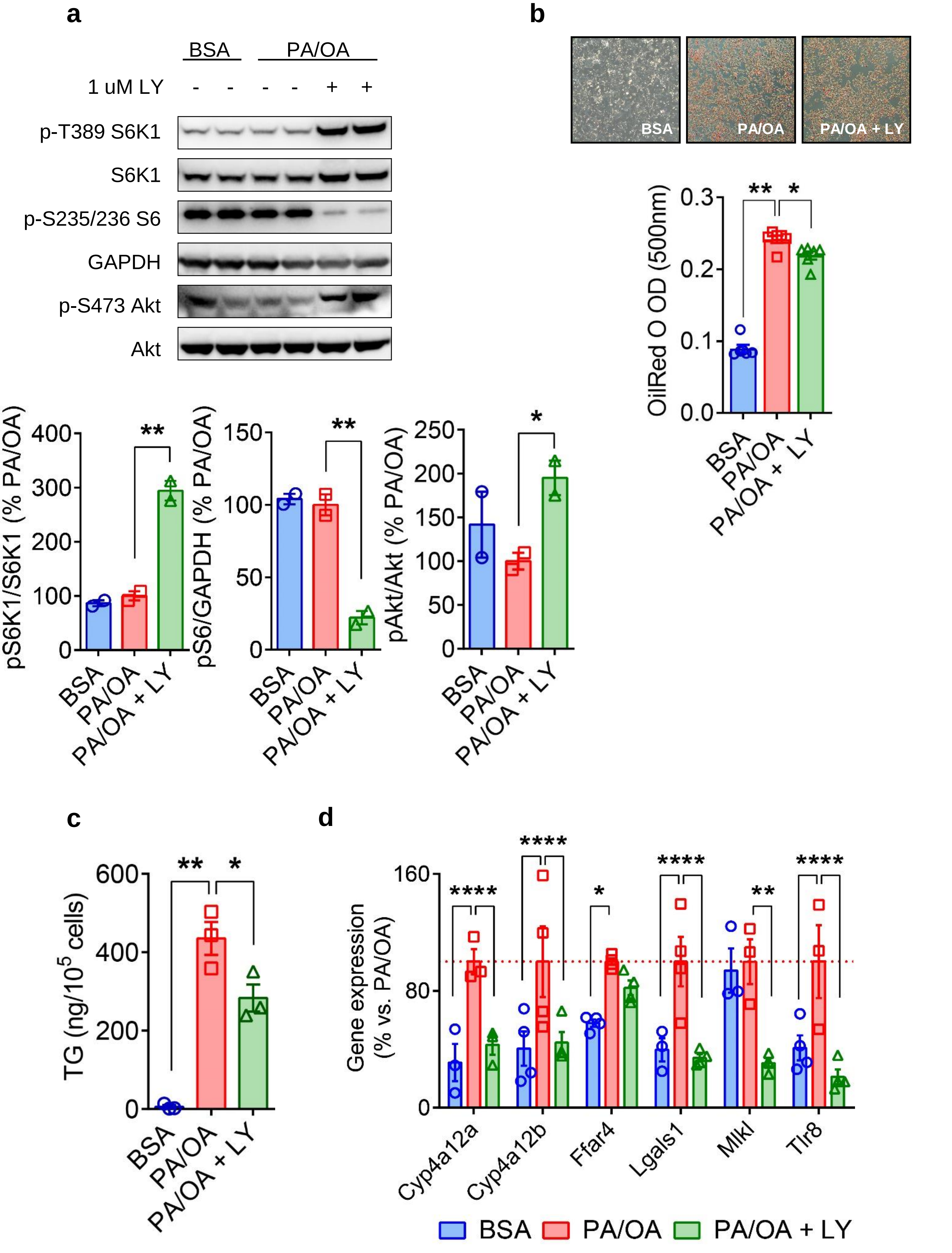

SUPPLEMENTAL FIGURE S4

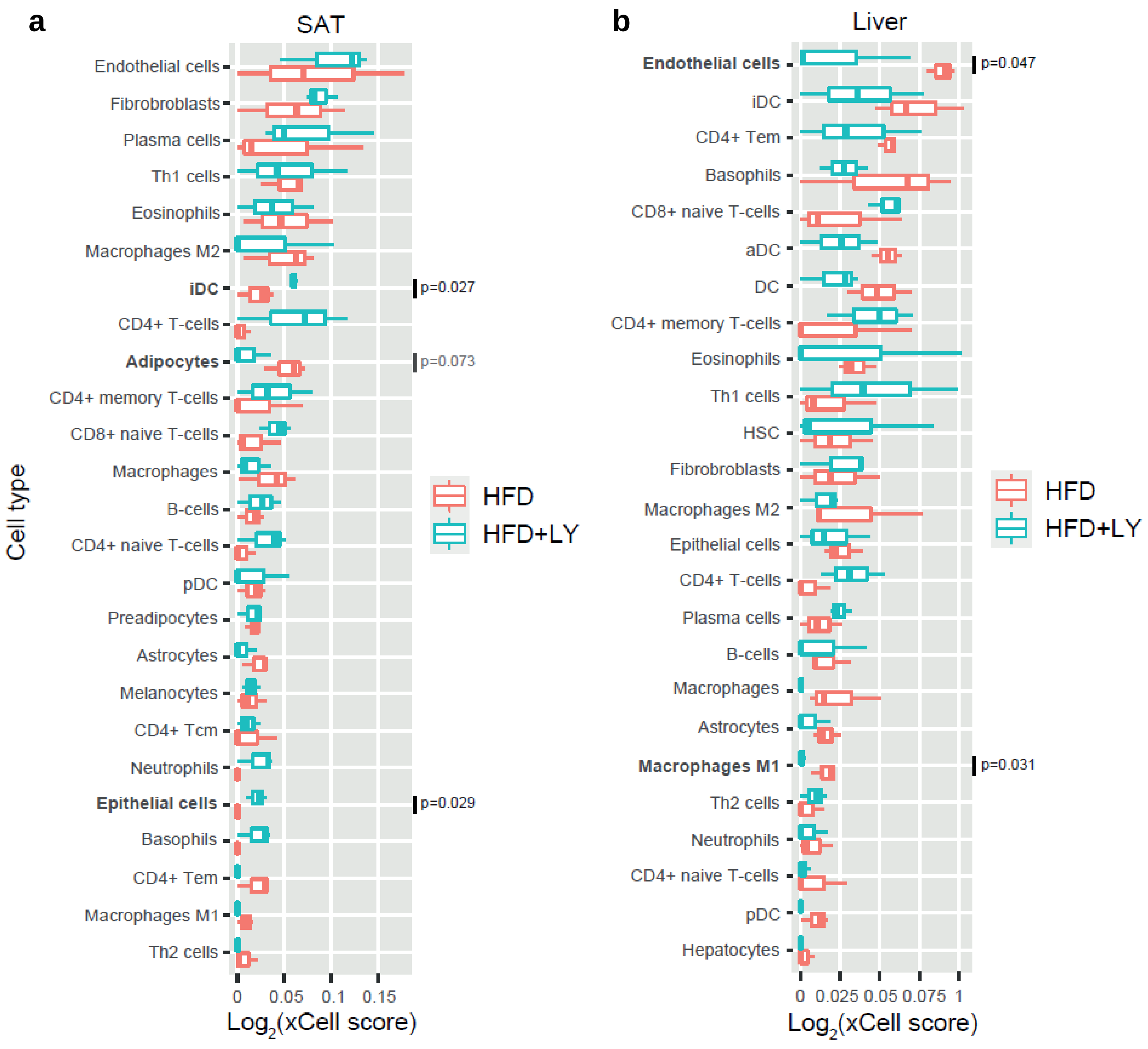

SUPPLEMENTAL FIGURE S5

a

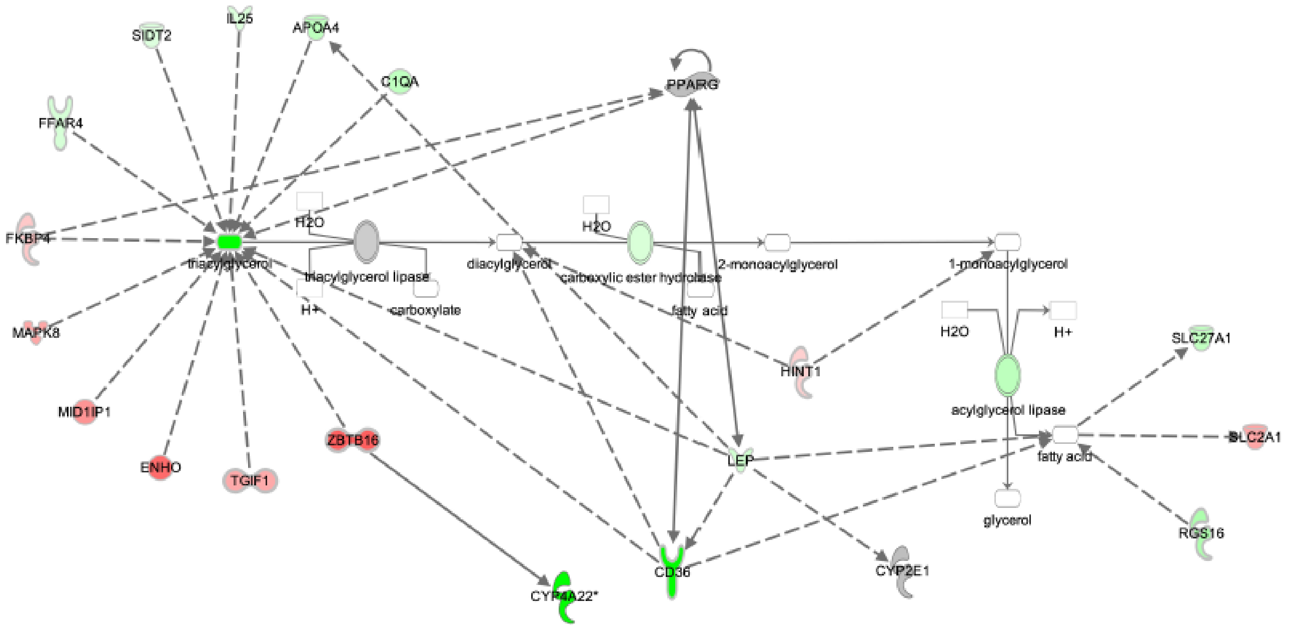

b

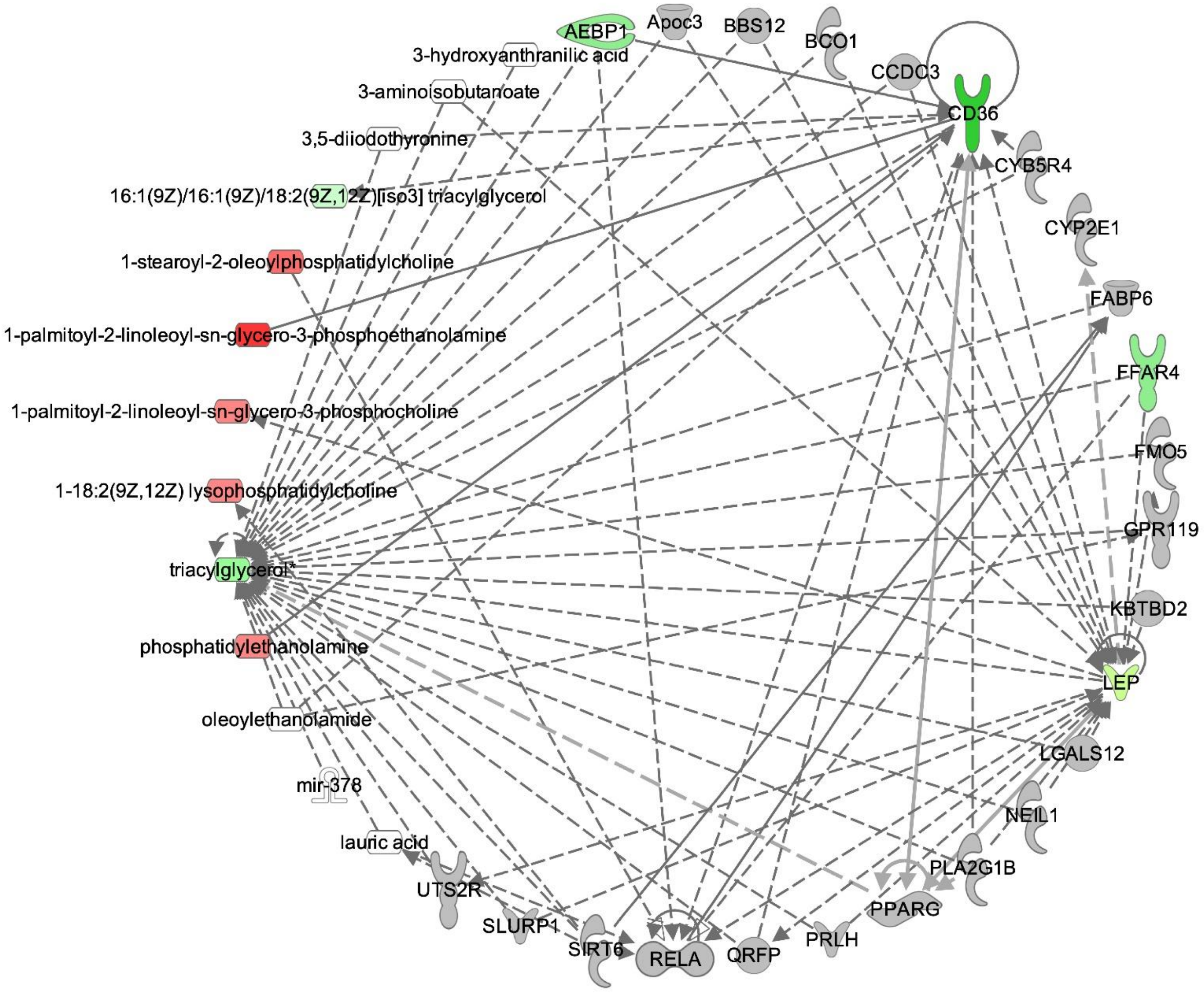

SUPPLEMENTAL FIGURE S6

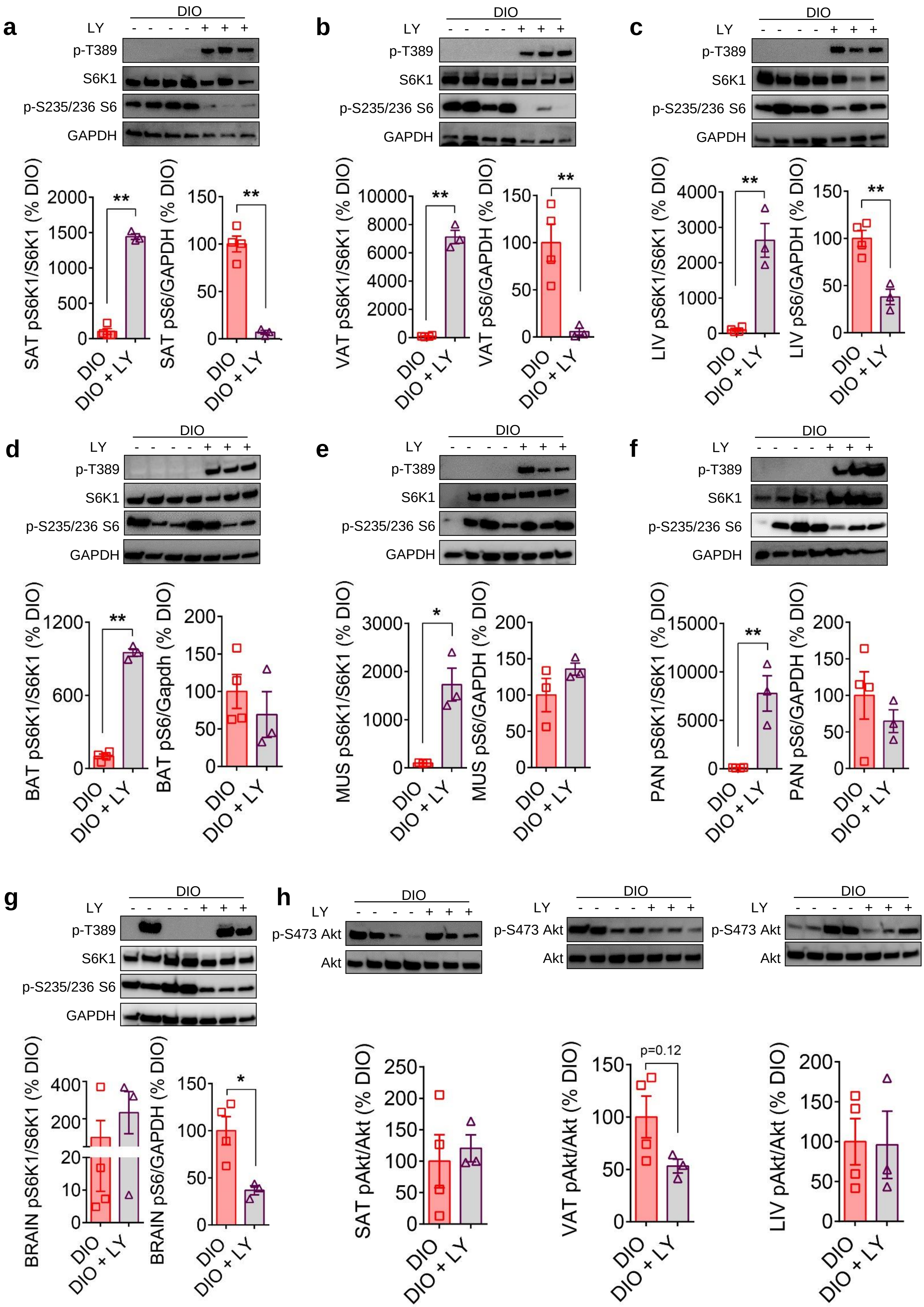

SUPPLEMENTAL FIGURE S7

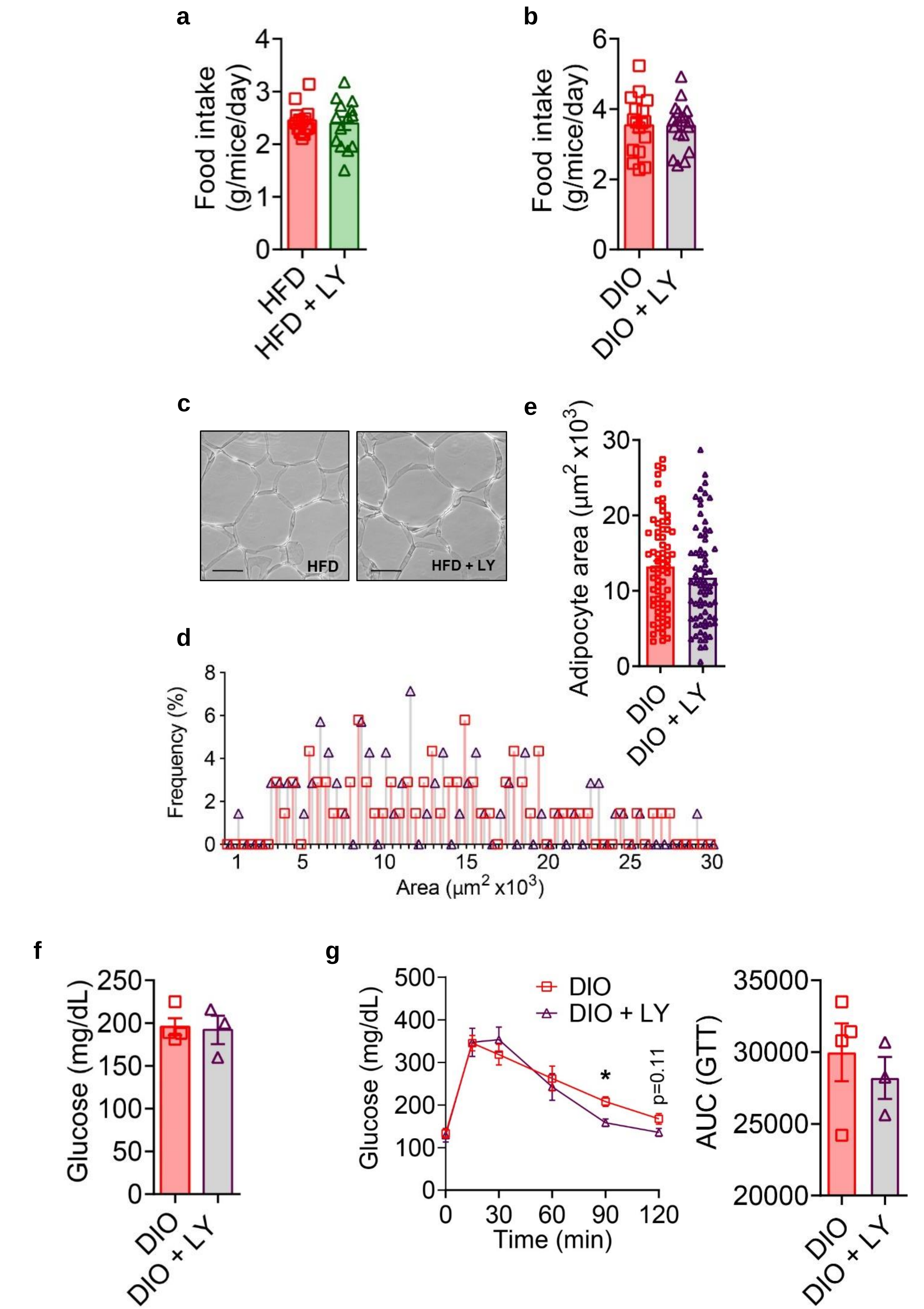

SUPPLEMENTAL FIGURE S8

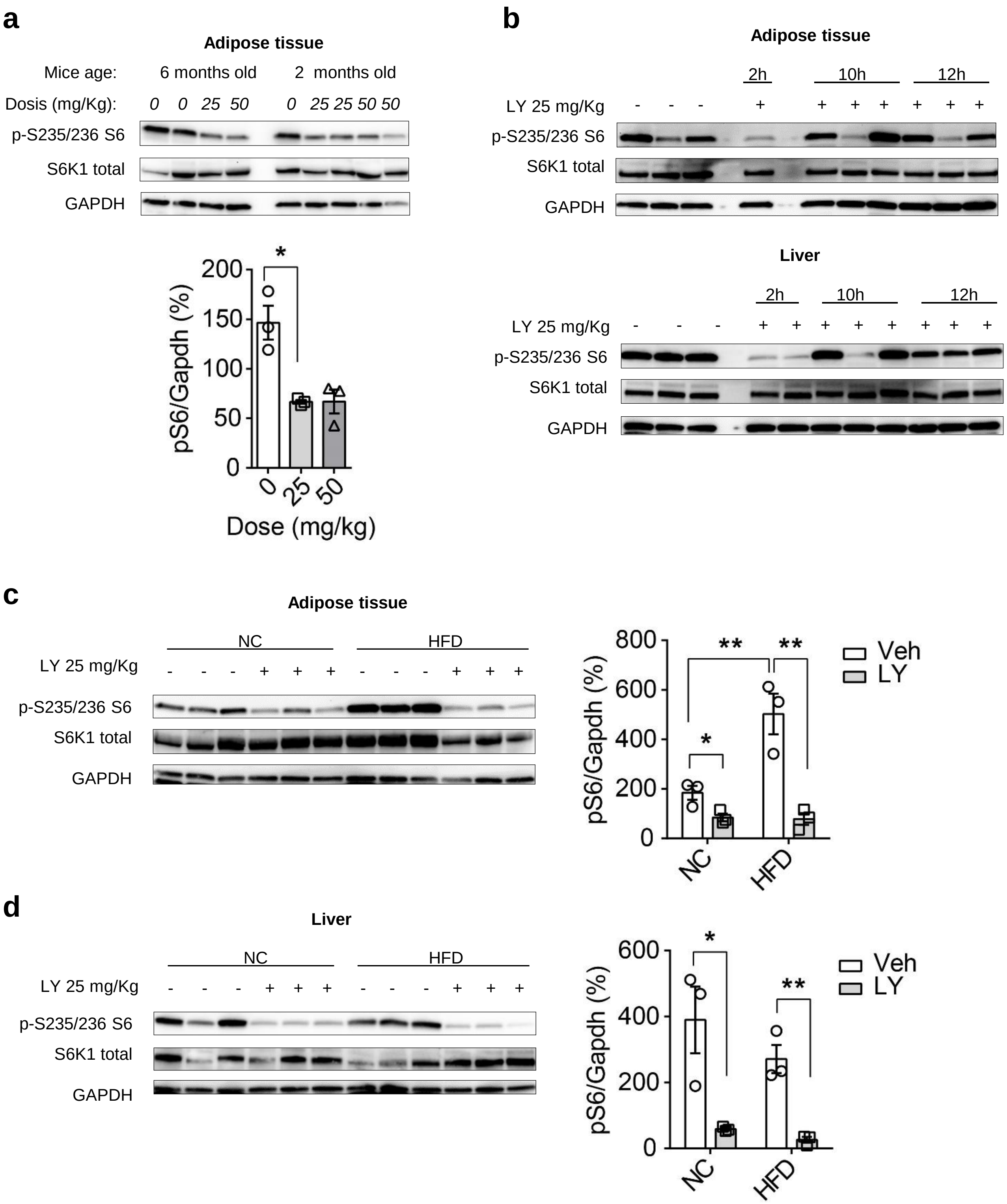

Supplement: Supplemental data [file jciinsight-7-150461-s083.pdf]
